# Endothelial transcriptomic, epigenomic and proteomic data challenge the proposed role for TSAd in vascular permeability

**DOI:** 10.1007/s10456-025-09971-x

**Published:** 2025-03-13

**Authors:** James T. Brash, Guillermo Diez-Pinel, Luca Rinaldi, Raphael F. P. Castellan, Alessandro Fantin, Christiana Ruhrberg

**Affiliations:** 1 UCL Institute of Ophthalmology, University College London, 11-43 Bath Street, London, EC1V 9EL UK; 2 Department of Biosciences, University of Milan, Via G. Celoria 26, 20133 Milan, Italy

**Keywords:** RNA-seq, Leaky gene expression, Active gene expression, VEGF, SH2D2A, TSAd, Endothelial cell, Vascular hyperpermeability

## Abstract

The vascular endothelial growth factor VEGF drives excessive vascular permeability to cause tissue-damaging oedema in neovascular and inflammatory diseases across multiple organs. Several molecular pathways have been implicated in VEGF-induced hyperpermeability, including binding of the VEGF-activated tyrosine kinase receptor VEGFR2 by the T-cell specific adaptor (TSAd) to recruit a SRC family kinase to induce junction opening between vascular endothelial cells (ECs). Inconsistent with a universal role for TSAd in permeability signalling, immunostaining approaches previously reported TSAd only in dermal and kidney vasculature. To address this discrepancy, we have mined publicly available omics data for expression of TSAd and other permeability-relevant signal transducers in multiple organs affected by VEGF-induced vascular permeability. Unexpectedly, TSAd transcripts were largely absent from EC single cell RNAseq data, whereas transcripts for other permeability-relevant signal transducers were detected readily. TSAd transcripts were also lacking from half of the EC bulk RNAseq datasets examined, and in the remaining datasets appeared at low levels concordant with models of leaky transcription. Epigenomic EC data located the TSAd promoter to closed chromatin in ECs, and mass spectrometry-derived EC proteomes typically lacked TSAd. By suggesting that TSAd is not actively expressed in ECs, our findings imply that TSAd is likely not critical for linking VEGFR2 to downstream signal transducers for EC junction opening.

**Supplementary Information:**

The online version contains supplementary material available at 10.1007/s10456-025-09971-x.

## Introduction

The vascular endothelial growth factor VEGF (VEGFA) is one of the most widely studied proteins [1] due to its crucial roles in cardiovascular development, cancer, ischemic disease and neovascular eye disease (e.g., Refs. [2–5]). VEGF promotes both blood vessel growth and vascular permeability, and VEGF upregulation therefore drives excessive vascular leakage and tissue-damaging oedema in neovascular and inflammatory diseases such as tumour ascites, diabetic retinopathy, age-related macular degeneration, stroke and myocardial infarction (e.g., Refs. [4, 6, 7]). Anti-VEGF therapies can be administered to attenuate pathological vascular leakage [5], but VEGF blockade is unsuitable when blood vessel growth should be stimulated, for example to promote the revascularisation of ischemic tissues. Accordingly, alternative therapeutic targets should be identified that are specific to the VEGF hyperpermeability signalling pathway without compromising beneficial VEGF-induced blood vessel growth in ischemic diseases. Much vascular biology research is therefore focussed on elucidating molecules that transduce VEGF signals in vascular endothelial cells (ECs) and could be targeted to modulate vascular permeability to fluids, solutes and larger molecules such as albumin [8–11].

To promote vascular hyperpermeability, VEGF binds the receptor tyrosine kinase VEGFR2 (also known as KDR or FLK1) and its co-receptor neuropilin 1 (NRP1) into a complex [12, 13]. In addition to NRP1, the proteoglycan syndecan 2 acts as a VEGFR2 co-receptor in the dermis and brain to segregate vascular permeability from angiogenic VEGF signalling [14]. Subsequent to VEGFR2 complex formation, SRC family kinase (SFK) signalling is activated, which sets in motion downstream pathways that target the junction protein vascular endothelial (VE-)cadherin (cadherin 5) in a process termed paracellular permeability [13, 15–19]. Agreeing with this model, mice globally deficient in either one of two SFKs, SRC or YES1, have an attenuated response to VEGF in vascular hyperpermeability assays [20], although another study reported that endothelial YES1 instead helps establish and maintain the vascular barrier [21].

The interaction between activated VEGFR2 and SRC has been proposed to be mediated by a third protein that binds the phosphorylated Y951 residue of VEGFR2 (Y949 in mice), the SH2 domain-containing T cell-specific adaptor protein (TSAd) that is encoded by the *SH2D2A* gene [22–26]. Supporting this model, VEGF-induced vascular leakage is reduced in mice expressing VEGFR2 with a Y949F mutation and in mice with a global TSAd deletion [24, 25]. However, TSAd was originally reported to be expressed in haemopoietic tissues and immune cells only [27], and an immunostaining-based survey of TSAd localisation in human tissues concluded that it was largely absent from blood vessels except in dermal vasculature [28]. Additionally, TSAd protein detection was reported in kidney vessels [22]. Nevertheless, endothelial-specific TSAd knockout mice remain to be examined for defects in vascular permeability to establish whether its role in VEGF-induced vascular permeability signalling is EC-autonomous.

In addition to the VEGFR2-TSAd-SRC axis, several other molecular pathways contribute to VEGF-induced hyperpermeability signalling through paracellular and/or transcellular routes, the former involving VE-cadherin-positive inter-endothelial junctions and the latter involving caveolae [9, 29, 30]. Amongst these pathways, signalling via the alternative VEGF receptor VEGFR1 and the caveolar protein caveolin 1 converges on the endothelial nitric oxide synthetase (eNOS), which nitrosylates beta-catenin and VE-cadherin, whereas the calcium-dependent AMPK/p38 MAP kinase pathway specifically enables paracellular permeability signalling downstream of VEGFR2 in the brain and retina, where they induce VE-cadherin rearrangements [9, 29–32]. Several intracellular kinases other than SRC also contribute to permeability signal transduction, including the ABL kinases ABL1 and ARG [13, 19] and the focal adhesion kinase FAK [33]. Vice versa, the vascular endothelial protein tyrosine phosphatase VE-PTP stabilises the endothelial barrier by targeting VE-cadherin phosphorylation and thus serves as a negative regulator of VEGF-induced hyperpermeability [34].

Basing the search for novel therapeutics to treat VEGF-induced vascular leakage on molecular interactions between any of the above or other signal transduction molecules obviously requires that the signalling partners are co-expressed in ECs of the organ to be treated. This prerequisite is readily testable with computational approaches that interrogate transcriptomic data of ECs from multiple organs yet needs to consider that transcripts for individual genes may be underrepresented in some types of datasets. For example, *SRC* transcripts are not readily detected in ECs of several publicly available sc-RNAseq datasets, but abundant in EC bulk RNAseq data [35]. Here, we have combined the analysis of publicly available sc-RNAseq, bulk RNAseq, epigenomic and proteomic datasets to compare the gene expression characteristics of signal transducers implicated in VEGF-induced vascular permeability signalling in ECs of several organs clinically affected by VEGF-driven oedema and in EC subtypes used for permeability studies in vitro. Our findings suggest that the *SH2D2A* gene encoding TSAd is not actively expressed in ECs of any of the organs examined, unlike all other signal transducers examined. This finding suggests that the current model of a VEGF-induced endothelial VEGFR2-TSAd-SRC complex needs to be revised to better support the search for novel therapeutics to reduce vascular hyperpermeability, and should be focussed on signal transducers expressed robustly in endothelial cells in vascular permeability-relevant organs.

## Methods

### Analysis of EC sc-RNAseq data

The human skin EC dataset was downloaded from the BIG Data Center (https://bigd.big.ac.cn/) [36]. The human trachea dataset was downloaded from the Human Cell Landscape project on GEO NCBI (https://www.ncbi.nlm.nih.gov/geo/) [37] and the EC cluster was selected according to expression of core endothelial markers such as *PECAM1*, as previously described [38]. We downloaded raw count data from the EC Atlas shiny app (http://endotheliomics.shinyapps.io/ec_atlas/) [39]. For the EC atlas and human datasets, we removed cells containing less than 200 feature counts. We downloaded *Tabula Muris* R objects containing gene expression data from cells isolated by fluorescence-activated cell sorting (FACS) or microfluidic droplet capture from http://figshare.com/articles/dataset/Robject_files_for_tissues_processed_by_Seurat/5821263; these Seurat objects had been pre-processed to remove cells containing less than 500 feature counts or less than 50,000 reads (FACS) and 10,000 UMIs (droplet), respectively [40]. EC clusters from brain, heart, lung and trachea obtained with the FACS isolation method were selected as described above. Moreover, ECs, T cells or natural killer (NK) cells were merged into one Seurat object per isolation method. R v4.4.1 [41] and Seurat 5.1.0 [42] versions were used to explore sc-RNAseq data. Downstream analysis included data normalisation (“LogNormalize” method and scale factor of 10,000) and variable gene detection (“vst” selection method, returning 2000 features per dataset). Principal components (PC) analysis (PCA) was performed on variable genes, and the optimal number of PCs was chosen for each sample using the elbow plot. The selected PCs were used for Louvain graph-based clustering. Uniform manifold approximation and projection (UMAP) was chosen as a non-linear dimensionality reduction method, and cluster cell identity was assigned by manual annotation based on known marker genes. Each relevant gene was then examined using the *FeaturePlot* and *VlnPlot* functions.

### Analysis of EC bulk RNAseq data

The Bulk-ECexplorer application for mining endothelial cell transcriptomic data (https://www.ruhrberglab.shinyapps.io/BulkECexplorer) compiles 240 high quality bulk RNAseq datasets from primary human ECs, human umbilical vein ECs (HUVECs) and human dermal microvascular ECs (HDMECs), and primary mouse ECs isolated from the lung, brain or retina [35]. We used the online interface of the Bulk-ECexplorer application to download graphs displaying expression data in TPM with or without a 1 TPM threshold, whereby the latter threshold is commonly used cut-off that was also validated to return results reflecting active gene expression in ECs [35]. We also used the online interface to download graphs returning validated predictions on active versus leaky gene expression, which are incorporated into the Bulk-ECexplorer application based on methods utilising Gaussian mixture models (GMM) and zTPM thresholds [35]. To evaluate gene expression correlations, *corr.test* from the ‘psych’ R package v2.4.3 was used to perform Pearson’s correlations (r) with Holm adjusted p-values, using zTPM values of genes with TPM > 0; *corrplot* from the ‘corrplot’ R package v0.92 and ‘ggplot2’ R package v3.5.0, were used to generate correlation plots. The relationship between the number of aligned reads and the probability of *SH2D2A* detection was assessed by logistic regression using the *glm* function in R v4.4.0. In our logistic regression model, the binary dependent variable represents the detection of *SH2D2A* (1 = detected, 0 = not detected) and the continuous predictor was the number of aligned reads. The final equation was logit(probability) = intercept + β * number of aligned reads =  − 0.97 + 3.96E-08 * number of aligned reads.

### Analysis of bulk RNA-seq samples from VEGF-stimulated ECs

The European Nucleotide Archive (ENA; https://www.ebi.ac.uk/ena/) was queried with the term ‘VEGF endothelial’, which yielded 339 different projects. From these, we selected three bulk RNA-seq datasets that contained both control and VEGF-stimulated HUVECs. Raw reads from projects PRJNA431557 and PRJNA945485 were downloaded from GEO (https://www.ncbi.nlm.nih.gov/geo/; accession numbers GSE109625 and GSE227549, respectively). Raw reads from project PRJNA807293 were downloaded from ENA (https://www.ebi.ac.uk/ena/). To obtain count matrices and TPM matrices, the nf-core/rnaseq v3.14.0 pipeline (https://zenodo.org/records/14537300) was run using Nextflow v24.04.3 [43] to align reads to the human reference genome Homo_sapiens.GRCh38.dna.primary_assembly.fa.gz with  the annotation file Homo_sapiens.GRCh38.113.gtf.gz (ENSMBL release 113). Differential expression analysis was carried out in R v4.4.1 using the DESeq2 v1.44.0 package [44].

### Analysis of the Encyclopaedia of DNA Elements (ENCODE) DNAse-seq database.

DNase I hypersensitive (HS) sites sequencing (DNase-seq) data from the ENCODE project were examined as meta-DNase tracks on the UCSC Genome Browser (http://genome.ucsc.edu/), as described [45]. The meta-DNase HS profile for ECs (n = 15) was comprised of HUVECs (n = 1), HDMECs (n = 10, including n = 4 blood vascular, n = 2 lymphatic vascular, n = 4 mixed blood and lymphatic vascular), lung ECs (n = 4, including n = 2 blood vascular and n = 2 lymphatic vascular). The meta-DNase HS profile for lymphoid samples (n = 15) was comprised of T cells (n = 12), natural killer cells (n = 1) and a B cell lymphoma line (n = 2). The meta-DNase HS profile for myeloid/erythroid samples (n = 15) was comprised of CD34 + erythroid (n = 8), myeloid (n = 3) and progenitor (n = 4) cells.

### Analysis of proteome datasets

Summary data of proteomic datasets were retrieved from the ProteomeXchange database (https://www.proteomexchange.org) in August 2024. To identify relevant endothelial datasets, we queried the archive for the terms ‘HUVEC’, ‘human umbilical cord endothelial’ and ‘endothelial cells’. Our queries returned 139 projects for either *Homo sapiens* or *Mus musculus*, which were individually examined to determine their suitability for our analysis. We only retained  published datasets for analysis that provided  detection values for individual proteins in pre-processed lists, for example Log_2_ of intensity Based Absolute Quantification (iBAQ) or label-free quantitation (LFQ). We included only datasets derived from whole cell lysates or which were enriched in the cytoplasmic and plasma membrane fractions (not secretome, exosome or nuclear fractions), and which detected at least one of the 2 core EC markers KDR or CDH5. As we wished to examine the ‘basal’ proteome of ECs, we excluded datasets from cells that had been stimulated (for example, with a small molecule or by hypoxia) and/or had been genetically or functionally modified (for example, by gene deletion, protein overexpression or immortalisation). However, we retained datasets in these projects that were derived from control cells (for example, vehicle-stimulated or small interfering RNA control-transfected ECs). The following 15 datasets comprised of 52 individual samples were retained and mined for detection of individual proteins: whole cell proteomes for HUVECs (PXD009687, PXD003284, PXD020958, PXD000359, PXD034985 and PXD045899), heart ECs (PXD026673 and PXD045899), lung ECs (PXD029834, PXD046787 and PXD045899), retina ECs (PXD005972), skin ECs (PXD019909), kidney ECs (PXD045899) and brain ECs (PXD045899) as well as cytoplasmic/plasma membrane protein-enriched samples from HUVECs (PXD003406). Additionally, we examined the 3 VEGF-stimulated samples from human lung ECs that were included in PXD029834. Moreover, we mined whole cell proteomes for T cells (PXD015872) as well as cytoplasmic/plasma membrane protein-enriched samples from normal human dermal fibroblasts (NHDFs; PXD003412) and peripheral blood-derived mononuclear cells (PBMCs; PXD001416). The cytoplasmic/plasma membrane protein-enriched samples were part of a larger project that compared HUVEC, NHDF and PBMC proteomes [46, 47].

To determine the total number of proteins detected in each dataset, we considered all proteins expressed by at least one sample in that dataset; missing values, zeroes and imputed intensity values of 0.001 for an individual protein were considered as ‘not detected’. In datasets lacking gene names, we mapped protein names or Uniprot accessions to their corresponding gene names using the Uniprot human reference proteome (https://www.uniprot.org/proteomes/UP000005640, accessed in October 2024). To assess the cellular signature of each sample, samples were examined for core EC markers (in addition to KDR or CDH5: FLT1, NOS3, VWF, PLXND1, TIE1, TEK, or PLVAP), immune cell (IC) markers (PTPRC, CD3E, CD247, CD8A, ITGAM) and mesenchymal cell (MC) markers (ELN, PDGFRA, PDGFRB, CD248). The  false discovery rate was q-value equal to 0.05. To assess cell type signatures of TSAd-expressing HUVEC proteomes from PDX003406, we first identified the proteins exclusive to the TSAd-expressing samples. Then, a hypergeometric test was carried out using the function *enricher* from the clusterProfiler v4.12.6 package to determine if any cell type signature gene sets from the Human Molecular Signatures Database were significantly enriched in the HUVEC samples that detected TSAd (file c8.all.v2023.2.Hs.symbols.gmt; https://www.gsea-msigdb.org/gsea/msigdb) [48]. For this, a list of all detected proteins across PDX003406’s EC samples was used as the background universe, and the Benjamini–Hochberg correction was applied to account for multiple testing.

## Results

### TSAd (SH2D2A) transcripts are not readily detected in ECs by multi-organ sc-RNAseq analysis

**Fig. 1:**
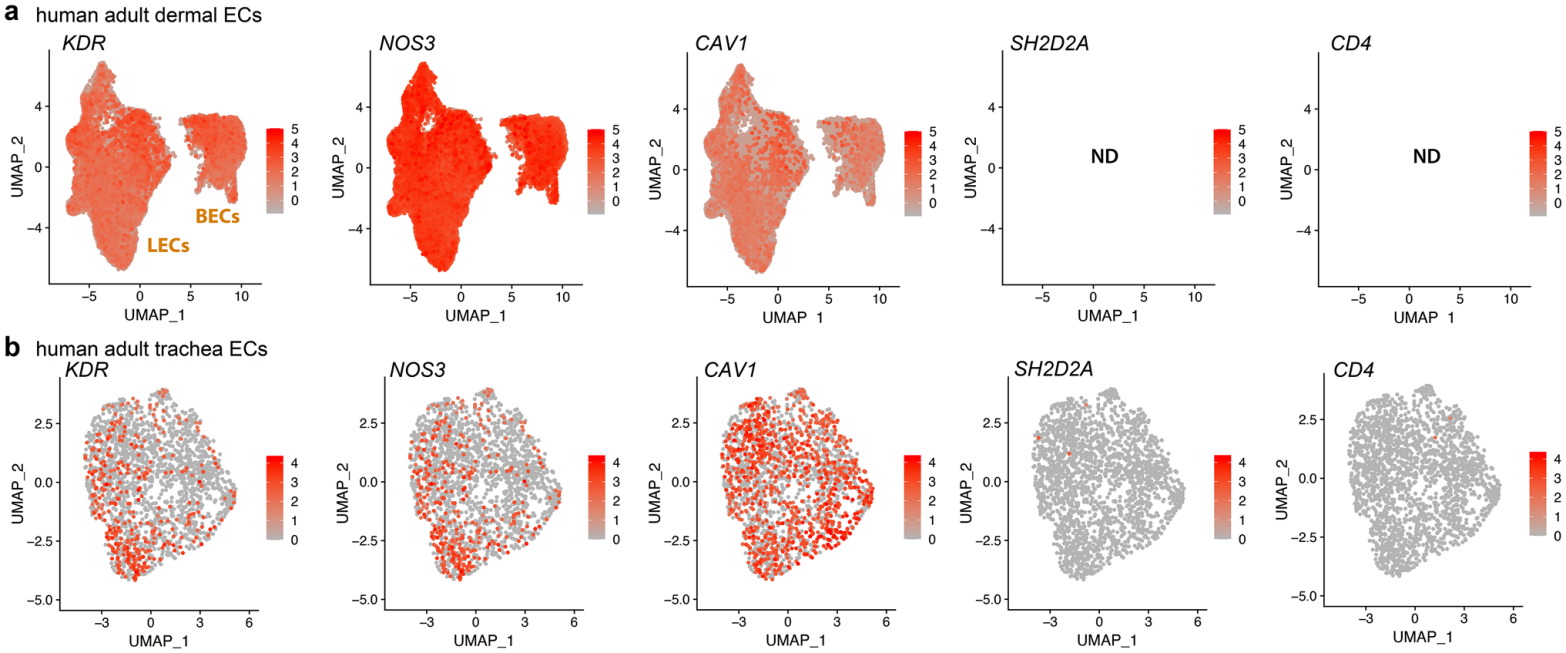
*SH2D2A* is rarely detected in sc-RNAseq data from human skin or trachea ECs. sc-RNAseq data from human adult skin after isolation of PECAM1 ECs by FACS (**a**) and from human adult trachea after EC identification and selection (**b**). UMAP projections were generated to compare expression levels for *KDR, NOS3*, *CAV1*, *SH2D2A* and *CD4*; each data point represents the value for a single cell. ND, not detected; BECs, blood ECs; LECs, lymphatic ECs

We initially used publicly available sc-RNAseq data to evaluate the expression of hyperpermeability-implicated genes in ECs. As the dermis has been widely used to investigate the VEGF hyperpermeability pathway via the Miles assay (e.g., [13, 24, 49–51], we first examined the expression of relevant signalling molecules in sc-RNAseq data of dermal ECs. FACS-isolated PECAM1-positive ECs from human adult dermis [36] form a cluster of blood vascular ECs and a smaller cluster of lymphatic vascular ECs [35]. Transcripts for the main EC markers and permeability pathway components VEGFR2 (*KDR*) and VE-cadherin (*CDH5*) as well as the key signalling mediators caveolin 1 (*CAV1*) and eNOS (*NOS3*) were readily detected in ECs of both clusters (Fig. 1a, Tables 1 and 2). We also detected all other permeability-relevant genes examined, including transcripts for the alternative VEGF receptors NRP1 (*NRP1*) and VEGFR1 (*FLT1*) and numerous signalling transducers/regulators implicated in VEGF hyperpermeability signalling [9, 29, 32], such as the vascular endothelial protein tyrosine phosphatase VE-PTP (*PTPRB*), syndecan 2 (*SDC2*), β-catenin (*CTNNB1*), AMPKA (*PRKAA1*), p38 (*MAPK14*), ABL1 (*ABL1*), ARG (*ABL2*) and focal adhesion kinase (*PTK2*) (Tables 1 and 2). *YES1* and *SRC* detection rates in this human dermis dataset have previously been described [35]. Unexpectedly, this dataset contained no UMI counts assigned to *SH2D2A* in any of its 47,668 EC transcriptomes (Fig. 1a, Tables 1 and 2). As a negative control, we examined dermal ECs for expression of *CD4*, an endothelial-inactive gene that is a key marker for CD4 + T cells and NK cells [52–54], two cell types that express *SH2D2A* [27, 28, 55]. As expected, dermal EC transcriptomes did not include *CD4* transcripts (Fig. 1a, Tables 1 and 2).

**Table 1:**
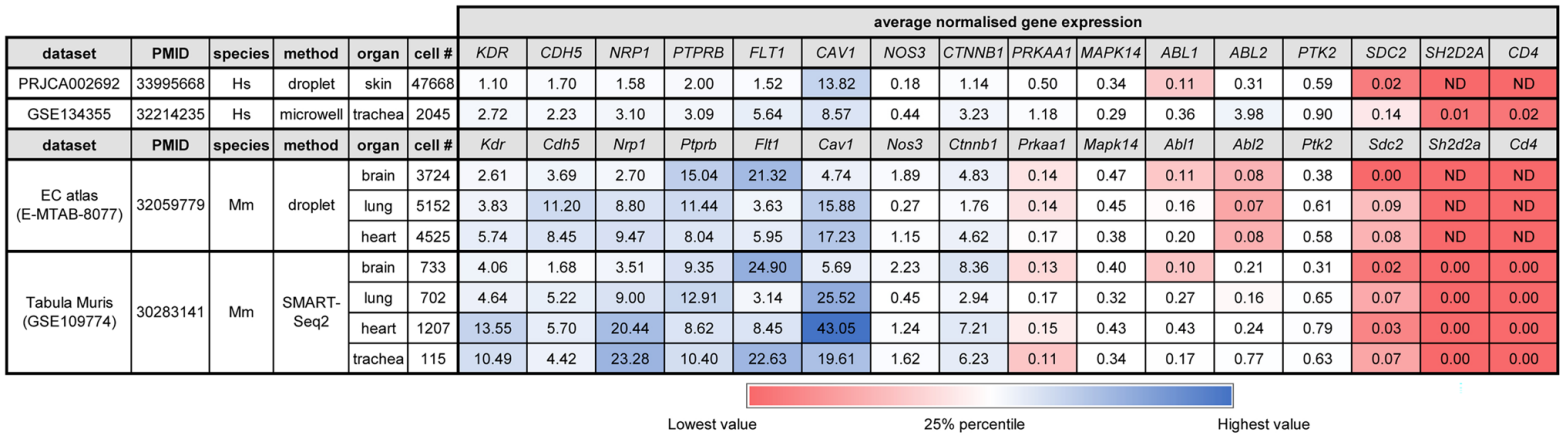
Average transcript levels for genes of the VEGF hyperpermeability pathway in ECs from sc-RNAseq datasets

Average transcript levels for genes of the VEGF hyperpermeability pathway and the non-EC, T cell marker *CD4* in ECs from the indicated organs in the indicated mouse and human sc-RNAseq datasets. EC selection was achieved either by FACS (E-MTAB-8077, PRJCA002692) or through clustering with Seurat (GSE109774, GSE134355). The table also lists the dataset name, accession number with Pubmed ID (PMID), species (Hs, *Homo sapiens* or Mm, *Mus musculus*), organ, method (droplet 10x, microwell or SMART-Seq2) and total EC number. Normalisation of gene expression was performed with Seurat. ND, not detected

**Table 2:**
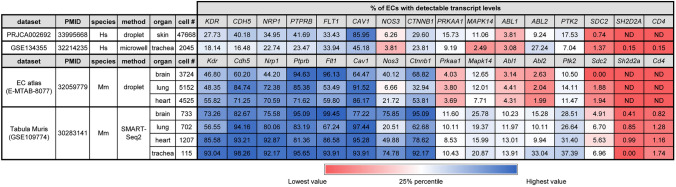
Prevalence of expression of genes in the VEGF hyperpermeability pathway in ECs from sc-RNAseq data

Percentage of ECs with detectable transcript levels for the indicated genes in the VEGF hyperpermeability pathway and the non-EC, T cell marker *CD4* in ECs from the indicated organs in the indicated mouse and human sc-RNAseq datasets. EC selection was achieved either by FACS (E-MTAB-8077, PRJCA002692) or through clustering with Seurat (GSE109774, GSE134355). The table also lists the dataset name, accession number with Pubmed ID (PMID), species (Hs, *Homo sapiens* or Mm, *Mus musculus*), organ, method (droplet 10x , microwell or SMART-Seq2) and total EC number. ND, not detected

**Table 3:**
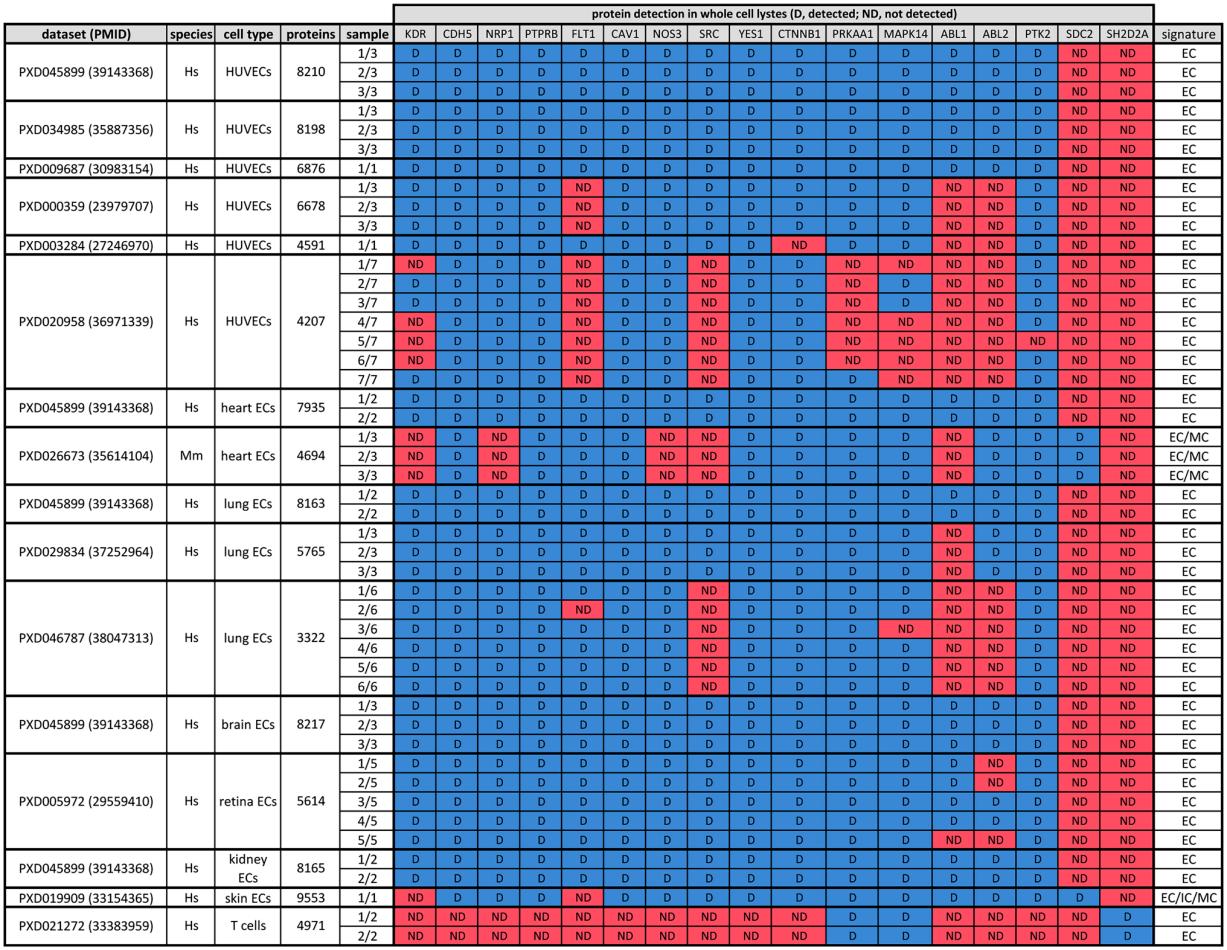
Detection of proteins in the VEGF hyperpermeability pathway

The table summaries whether the indicated proteins were detected (D) or not detected (ND) in 16 publicly available human and mouse datasets obtained by mass-spectrometry of whole cell lysates. Only samples from healthy, untreated conditions were scored; HUVEC data were derived from cultured cells. The table also lists the ProteomeXchange identifiers and Pubmed ID (dataset), species (Hs, *Homo sapiens* or Mm, *Mus musculus*), cell type, including EC subtype, number of detected proteins (proteins), number of independent samples per dataset (sample) and whether the proteome includes EC, immune cell (IC) and/or mesenchymal cell (MC) markers (cell type signature defined by key markers presented in Supplementary Table 3)

Lack of *SH2D2A* detection in dermal ECs was unexpected, considering that a prior study reported TSAd protein in dermal vasculature [27] and another study reporting that TSAd knockout mice lack a VEGF response in the Miles assay [24]. As the trachea is well suited to investigate vascular hyperpermeability pathways [56] and has also been used to implicate TSAd in permeability signalling [24], we next analysed adult sc-RNAseq data from ECs in the human trachea via the Human Cell Landscape compendium [37]. *YES1* and *SRC* detection rates have already been described for this dataset [35]. Transcripts for *KDR, CDH5, CAV1, NOS3* and all other examined genes implicated in the VEGF hyperpermeability were readily detected, whereas *SH2D2A* was detected in only 3/2045 trachea ECs with very low average transcript levels (Fig. 1b, c, Tables 1 and 2). This low detection level and frequency was again similar to that of *CD4*, which was detected at low levels in 2/2045 trachea ECs (Fig. 1b, c, Tables 1 and 2).

Next, we analysed sc-RNAseq data from the mouse, which has been the main model organism employed to define the roles of molecules in the VEGF hyperpermeability pathway (e.g., Refs. [12, 13, 17, 24, 33, 49, 50, 57]). Specifically, we took advantage of two sc-RNAseq compendia that contain a large number of ECs each, the EC atlas [39] and *Tabula Muris* [40]. The EC atlas contains data from ECs isolated with a combined MACS and FACS pipeline from 11 adult organs, whereas *Tabula Muris* contains data from ECs isolated with a FACS pipeline or droplet method from 10 adult organs. Notably, both compendia included the brain, lung and heart as examples of organs in which VEGF-induced vascular hyperpermeability has been observed.

In both the EC atlas and *Tabula Muris*, transcripts for *Kdr* and *Cdh5* were readily detected in the brain, lung and heart EC populations (Tables 1 and 2; *Yes1* and *Src* detection rates have been described [35]). ECs from the brain, lung and heart in both compendia also contained abundant transcripts for *Cav1*, *Nrp1*, *Flt1*, *Ctnnb1* and *Ptprb* (Tables 1 and 2). Transcripts for other known signal transducers in the VEGF hyperpermeability pathway were also detectable, although less frequently and at more moderate levels, including *Nos3*, *Sdc2*, *Ptk2*, *Prkaa1*, *Mapk14*, *Abl1* and *Abl2* (Tables 1 and 2). By contrast, no UMI counts assigned to *Sh2d2a* were identified with the EC atlas in brain, lung and heart ECs, and both its detection level and detection rate were very low with *Tabula Muris* in these organs (Tables 1 and 2; *Tabula Muris* detection rate: brain ECs 3/733, lung ECs 6/702, heart ECs 12/1207). *Tabula Muris* also contains data for the mouse trachea, which was studied to put forward a role for TSAd in the VEGF hyperpermeability pathway [24]; however, *Sh2d2a* transcripts were also lacking from trachea ECs (Tables 1 and 2).

**Fig. 2:**
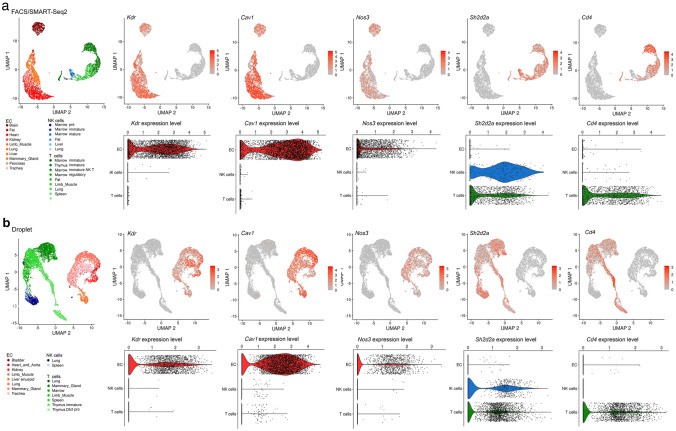
*Sh2d2a* is rarely detected in EC sc-RNAseq data from *Tabula Muris. Tabula Muris* sc-RNAseq data for the indicated genes, shown for FACS-captured (**a**) and droplet-captured (**b**) cells from pooled EC and immune clusters. A colour coded UMAP projection of cell type and tissue of origin is shown alongside UMAP projections illustrating the expression levels of the indicated genes per cell in each cluster. The corresponding violin plots illustrate the expression levels or each cell cluster. Each data point represents the value for one cell

Next, we extended analysis of *Sh2d2a* transcripts to all ECs across all organs in the EC atlas; however, *Sh2d2a* transcripts were again not detected. Further, we analysed the entire pool of FACS-isolated ECs across all 10 *Tabula Muris* organs alongside pooled T cell and NK cells, which are known to express *Sh2d2a* (Fig. 2a). Moreover, we performed a similar, parallel analysis of the entire pool of ECs isolated with the alternative droplet method from 9 adult organs (Fig. 2b). We again found that *Sh2d2a* transcripts were rarely detected in ECs, i.e., in only 1% of FACS-captured ECs (39/4155) and in only 0.5% of droplet-captured ECs (18/3725) of *Tabula Muris*. Moreover, ECs with detectable *Sh2d2a* were as rare as ECs with detectable *Cd4* (Fig. 2a, b), and average *Sh2d2a* transcript levels in ECs were as low as those for *Cd4* (Fig. 2a, b). Low *Sh2d2a* detection rates and low transcript levels in ECs could not be attributed to technical difficulties in identifying *Sh2d2a* transcripts by sc-RNAseq, because *Sh2d2a* transcripts were readily detected in NK cells and T cells by both the FACS and droplet method in *Tabula Muris* (Fig. 2a, b). Vice versa, and as expected, the EC marker *Kdr* was not detected in NK cells or T cells (Fig. 2a, b). These observations on mouse and human organ ECs can be confirmed by directly exploring the publicly available interactive webtools of the original sc-RNAseq datasets (EC atlas: https://endotheliomics.shinyapps.io/ec_atlas/; *Tabula Muris*: https://tabula-muris.sf.czbiohub.org/visualizations; Human Cell Landscape: https://db.cngb.org/HCL/landscape.html).

Taken together, the above transcriptomic analyses suggest that TSAd transcripts are either not expressed in ECs or expressed at levels that are not readily detectable by sc-RNAseq. We therefore complemented sc-RNAseq analysis with bulk RNAseq analysis.

### TSAd (*SH2D2A*) transcripts are absent or detected at low levels in EC bulk RNAseq data

Bulk RNAseq has an excellent capacity for detecting lowly expressed genes in homogenous cell populations [58]. For example, *SRC* is not easily detectable in sc-RNAseq datasets from mouse and human organ ECs but readily detected across most of the 240 mouse and human endothelial bulk RNAseq datasets included in the Bulk-ECexplorer compendium [35]. Relevant for the present investigation, the Bulk-ECexplorer datasets are derived from EC subtypes that have been used to characterise the VEGFR2-TSAd-SRC pathway, namely human dermal microvascular ECs (HDMECs) [13, 24], human umbilical vein ECs (HUVECs) [22, 59], mouse lung ECs [13, 25] and mouse retina ECs [26]. The Bulk-ECexplorer also contains datasets from ECs of the mouse brain, an organ in which VEGF-induced vascular leakage is a significant concern [60]. Moreover, transcripts for the permeability-relevant EC markers *KDR* and *CDH5* are defining features of the Bulk-ECexplorer datasets. To analyse the gene expression characteristics of permeability-relevant genes in ECs, we therefore took advantage of the Bulk-ECexplorer application, hosted at https://ruhrberglab.shinyapps.io/BulkECexplorer [35].

**Fig. 3:**
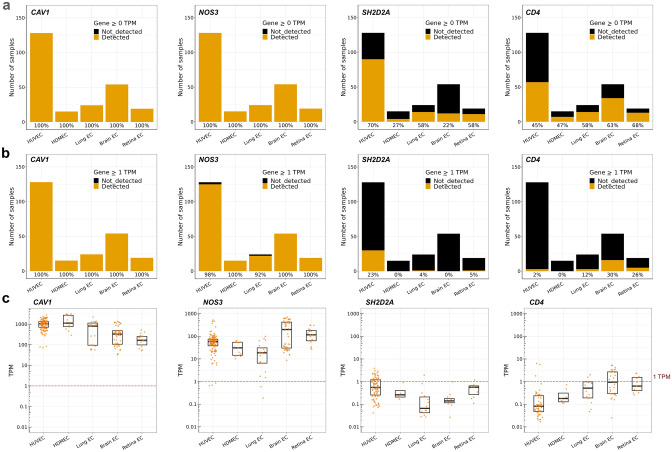
Bulk RNAseq reveals absent or low *SH2D2A* expression in human and mouse ECs. Expression of *CAV1*, *NOS3, SH2D2A* and *CD4* in endothelial bulk RNAseq datasets (HUVEC n = 128, HDMEC n = 15, mouse lung EC = 24, mouse brain EC = 54, mouse retina EC n = 19). **a, b** Stacked bar charts depict the total number of bulk RNAseq datasets analysed and the frequency at which transcripts for the indicated genes were (**a**) detected (> 0 TPM) versus not detected (= 0 TPM), or (**b**) detected (≥ 1 TPM) versus not detected (< 1 TPM) above the 1 TPM threshold; data are resolved by EC subtype. **c** Transcript levels for the indicated genes with expression > 0 TPM in each dataset for the indicated EC subtypes, including boxplots to illustrate the median and interquartile range; each data point represents one dataset. The dashed red line indicates the 1 TPM threshold. For n, see corresponding source data file

Similar to transcripts for the vascular barrier regulators *YES1* and *SRC* [35], transcripts for other permeability-relevant genes were detected by the Bulk-ECexplorer at high frequency (Figs. 3a, S1A, Supplemental Table 1). Thus, transcripts for *CAV1* and *NOS3* were detected in all 240 EC RNAseq datasets of the Bulk-ECexplorer (Fig. 3a), and also for all permeability signalling regulators examined, including *NRP1*, *FLT1*, *PTPRB*, *SDC2, PTK2*, *CTNBB1*, *PRKAA1*, *MAPK14*, *ABL1* and *ABL2* (Supplemental Fig. 1). By contrast, *SH2D2A* transcripts were not detected in 45% of the 240 EC datasets in the Bulk-ECexplorer (Fig. 3a; Supplemental Table 1). *SH2D2A* detection in the other 55% of the EC datasets was mostly in HUVECs, the patho-physiologically least relevant EC subtype for vascular permeability studies amongst the five EC subtypes included in the Bulk-ECexplorer (e.g., *SH2D2A* detection in 27% of HDMEC versus 70% of HUVEC datasets; Fig. 3a). Moreover, *SH2D2A* transcripts, when present, were typically detected at very low levels, within a TPM range that was orders of magnitude smaller than the TPM range for *CAV1*, *NOS3* or other permeability-relevant signal transducers (Figs. 3c; S1B). We therefore applied the 1 TPM threshold as a commonly used cut off for selecting genes suitable for downstream analysis, and thus found that only 13% of the 240 EC datasets in the Bulk-ECexplorer scored positive for *SH2D2A*, and these datasets were again almost exclusively derived from HUVECs (Fig. 3b).

Insufficient read depth can sometimes explain the absence of a transcript in bulk RNAseq data [61]. In agreement, *SH2D2A* detection in the 240 datasets of the Bulk-ECexplorer positively correlated with higher read depth (Supplemental Fig. S2). Nevertheless, genes detectable only at high read depth are naturally expressed at low levels [62]. Further, we considered that expression levels below 1 TPM in only a subset of EC samples is also observed for numerous genes not expected to be functional within ECs, for example, several ocular genes (*LENEP*, *CRYBB2*) and sex cell-specific genes (*DDX4*, *GDF9, YBX2, SPACA4*) [35]. We therefore compared the expression characteristics of *SH2D2A* to those of the T cell marker *CD4*, which is not expected to be expressed in ECs and is only rarely detected in EC sc-RNAseq data (see Fig. 2). *CD4* was detected in nearly half of the EC datasets included in the Bulk-ECexplorer (48% detection frequency), but only 11% of EC datasets expressed *CD4* at levels above 1 TPM (Fig. 3b, data resolved per EC type). When *CD4* expression was detected in ECs, it was within a TPM range orders of magnitude smaller than the TPM range for *CAV1, NOS3* or other permeability-relevant signal transducers, as observed for *SH2D2A* (Figs. 3c, S1B). Therefore, TSAd transcripts were either not detected in EC bulk RNAseq datasets or were detected at very low levels, akin to an EC-inactive gene.

### TSAd (*SH2D2A*) transcript levels in ECs are not upregulated in response to VEGF

To assess whether *SH2D2A* expression was increased upon VEGF stimulation, we performed differential expression analyses of three published bulk RNAseq data, which we identified by searching the European Nucleotide Archive (ENA; https://www.ebi.ac.uk/ena/) for the term ‘VEGF endothelial’. We identified three HUVEC datasets that contained both control and VEGF-stimulated samples. Amongst these, two different projects [63, 64] stimulated HUVECs cultured in 2D with VEGF and analysed them at time points from 1 to 12, or at 24 h post treatment, respectively. Among the VEGF-induced hyperpermeability pathway members, *FLT1* appeared upregulated from 1 to 24 h and *CTNNB1* from 1 to 12 h after VEGF exposure (Supplemental Table S2). *ABL2* and *YES1* appeared transiently upregulated 1 h after VEGF treatment, and *CAV1*, *KDR* and *NRP1* transiently 4 h after VEGF treatment (Supplemental Table S2). In another project, HUVECs were grown as spheroids to model sprouting angiogenesis [65]. *FLT1*, *CTNNB1* and *NRP1* were upregulated 18 h after VEGF exposure (n = 5 per group). By contrast, transcript levels of the other permeability pathway genes did not appear to be significantly enriched in any of the datasets (Supplemental Table S2). As observed for HUVEC datasets in the Bulk-ECexplorer, all permeability pathway genes examined were detected robustly across all three datasets, with exception of *SH2D2A*, whose TPM values ranged from 0 to 1 TPM in 14/14 control samples and in 12/14 VEGF-stimulated samples, and was detected at 1 or 2 TPM in the remaining two samples (Supplemental Table S2).

### Low TSAd (*SH2D2A*) levels in ECs are predicted to arise from leaky gene expression

Studies across several different cell types have found that lowly expressed (LE) transcripts can arise from leaky transcription, a phenomenon in which highly expressed (HE) genes impart a ‘transcriptional ripple effect’ on nearby genes that lack active chromatin markers [62, 66–69]. Notably, such LE genes do not usually give rise to detectable protein, and their transcripts are therefore thought to be biologically irrelevant [70–72]. Like other cell types, ECs contain a mixture of LE and HE transcripts whose combined expression values assume a bimodal distribution [35]. To predict whether transcripts arise from actively and leakily expressed genes, a two component Gaussian mixture model (GMM) can be fitted to such gene expression data and the probability of genes belonging to either the LE or HE distribution calculated, as initially done to examine the T cell transcriptome [73, 74], and subsequently applied to each one of the 198 bimodally distributed EC datasets included in the Bulk-ECexplorer [35].

**Fig. 4:**
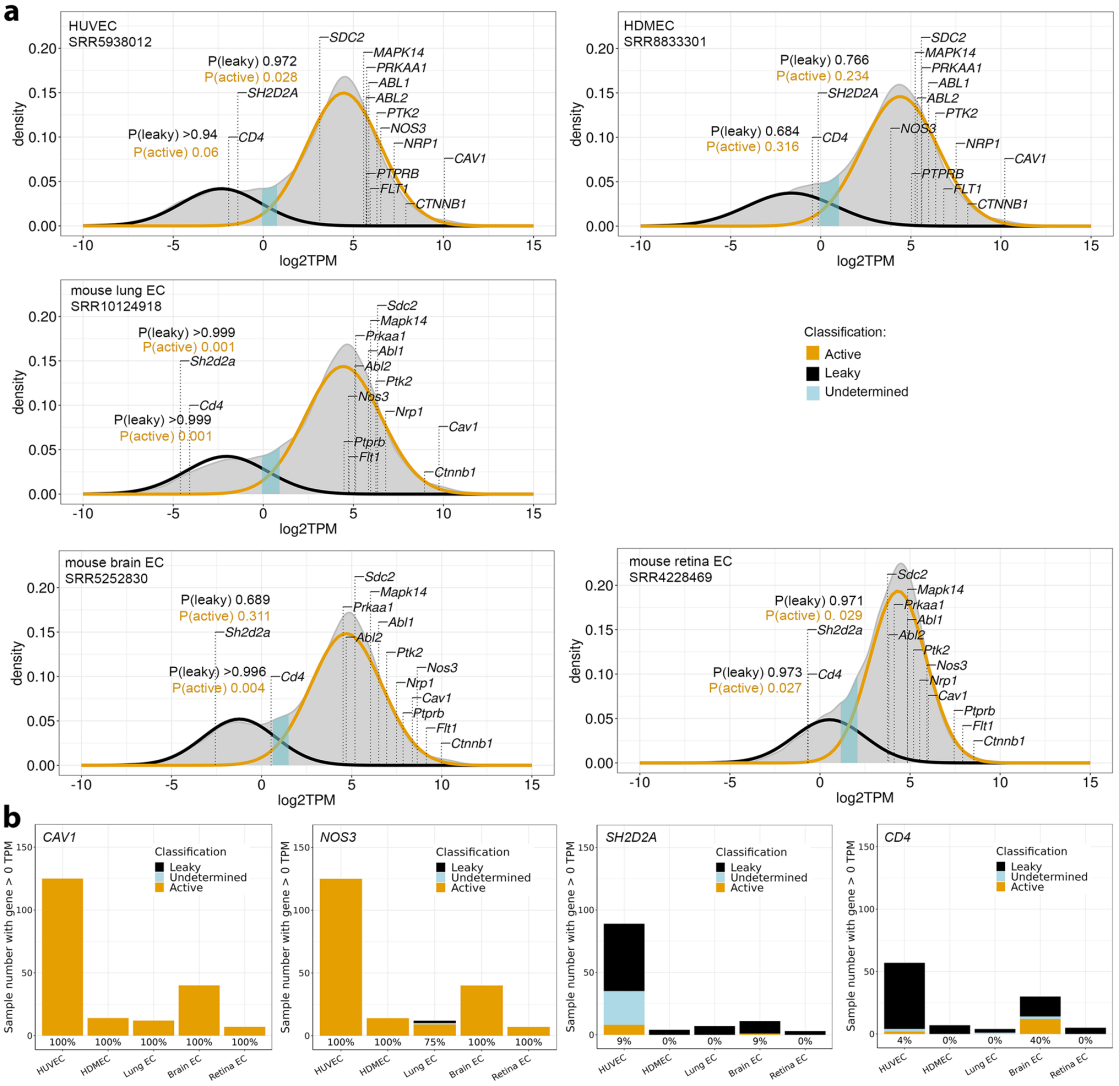
GMM-based classification links *SH2D2A* detection to leaky transcription. **a** Illustrative kernel density estimates of log_2_-transformed TPM values for protein-coding genes in the indicated bulk RNA seq datasets from the Bulk-ECexplorer. The expectation maximisation algorithm was used to estimate the parameters of a low Gaussian distribution (predicted leaky expression, black line) and a high Gaussian distribution (predicted active expression, gold line). Specific log_2_TPM values are shown for known vascular permeability signal transducers as well as *SH2D2A* and *CD4*. The P(active) and P(leaky) values are shown for *SH2D2A* and *CD4* and for all indicated genes listed in Supplemental Table 1. **b** Stacked bar charts show the number of Bulk-ECexplorer datasets for each EC subtype in which the indicated genes were classified with the GMM method as actively expressed (gold), leakily expressed (black) or in which the gene was not classified (undetermined, blue). Only datasets with a bimodal distribution of gene expression values analysed. Number of bimodally distributed datasets with detectable transcript for the examined gene: *CAV1* and *NOS3* n = 198; *SH2D2A* n = 114, *CD4* n = 103. The percentage of datasets in which each gene was classified as actively expressed is reported for each EC subtype below each bar

Similar to *SRC* and *YES1* [35], *CAV1* and *NOS3* were classified by the GMM approach as actively expressed in the vast majority of eligible (i.e., bimodal) Bulk-ECexplorer datasets in which their transcripts were detected (> 98%), and similar results were obtained for *NRP1*, *FLT1*, *PTPRB*, *SDC2, PTK2*, *CTNBB1*, *PRKAA1*, *MAPK14*, *ABL1* and *ABL2* (Figs. 4, S3 and Supplemental Table 1). Amongst the permeability regulators examined, two exceptions were *SDC2* and *SH2D2A. SDC2* was classified as actively expressed in 84% of datasets in which it was detected (164 out of 196), whereas *SH2D2A* was classified as actively expressed in only 8% of datasets in which it was detected (9 out of 114). When resolved by EC subtype, *SDC2* was predicted to be actively expressed in most HUVEC and lung EC datasets; furthermore, it was never classified as leakily expressed in datasets from brain, retina or dermal ECs, the organs in which SDC2 was reported to regulate vascular permeability signalling [14]. By contrast, *SH2D2A* was classified as actively expressed more often in HUVECs than in EC subtypes of vascular permeability-relevant organs (Fig. 4). Notably, *CD4* was classified as actively expressed at a frequency similar to *SH2D2A* (14% of 103 samples in which transcripts were detected; Fig. 4). GMM classification therefore predicted that all signalling molecules examined were expressed at functional levels in ECs of vascular permeability-relevant organs, except TSAd, which is classified as leakily expressed when transcript is detectable at all, similar to the EC inactive *CD4* gene.

**Fig. 5:**
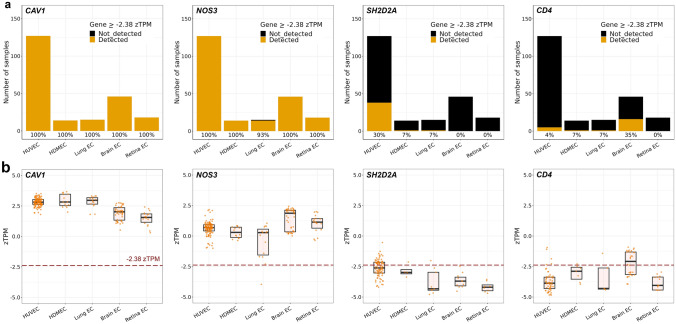
zTPM standardisation links *SH2D2A* detection in ECs to leaky transcription. Number of datasets analysed across EC subtypes for each of the four genes n = 220. The active expression threshold of -2.38 zTPM was previously determined for HUVEC. **a** Stacked bar charts show the number of datasets for each EC subtype in which the indicated genes were detected above (gold) or below (black) the -2.38 zTPM threshold. The percentage of datasets in which the indicated gene was expressed above the -2.38 zTPM threshold is reported below each bar for the corresponding EC subtype. HUVEC n = 127, HDMEC n = 14, mouse lung EC = 15, mouse brain EC = 46, mouse retina EC n = 18. **b** Range of zTPM values for the indicated genes in each of the indicated EC subtypes, including boxplots to illustrate the interquartile range. Each data point represents one dataset; values are shown together with boxplots to illustrate the interquartile range (for TPM values and n, see corresponding source data file). The dashed red line indicates the -2.38 zTPM threshold. Only datasets with gene detection > 0 TPM and without a unimodal distribution were included

We next used the zTPM tool in the Bulk-ECexplorer as an alternative method to predict whether a gene is actively or leakily expressed [35]. This tool applies a zTPM transformation to each of 220 datasets in the Bulk-ECexplorer without unimodal expression distribution before determining whether a queried gene exceeds the predicted zTPM threshold for actively expressed genes [35]; this threshold was previously determined based on HUVEC epigenomic and RNAseq data from the Encyclopaedia of DNA Elements (ENCODE) project [62]. Using this method, we found that VEGF hyperpermeability-relevant genes examined exceeded the threshold for actively expressed genes in the vast majority of datasets that contained their transcripts (> 92%), with exception of *SH2D2A*, which exceeded the threshold in only 18% of the 220 datasets, and these were again mostly derived from HUVECs (Figs. 5, S4, Supplemental Table 1). *CD4* transcript levels similarly exceeded the threshold in only 11% of the 220 datasets. zTPM-based classification therefore agrees with GMM-based classification to predict that all signalling molecules examined are expressed at functional levels in ECs of vascular permeability-relevant organs, except TSAd, which instead is expressed like a leaky gene, similar to the EC inactive gene *CD4*.

Taken together, *SH2D2A*’s gene expression characteristics in ECs were similar to those of a typical non-EC gene and therefore inconsistent with the prediction of functional gene transcription in ECs.

### *SH2D2A*’s genomic position suggests susceptibility to a transcriptional ripple effect

In immune cells, LE genes often have HE genomic neighbours, with the distance of HE genes to the nearest LE gene typically smaller than the distance of HE genes to the nearest not expressed (NE) gene [67]. We therefore asked whether low *SH2D2A* transcript levels in ECs correlated with the gene’s localisation near HE genes that might confer a transcriptional ripple effect on *SH2D2A*. Thus, we first examined gene expression characteristics in *SH2D2A’s* genomic neighbourhood and then correlated *SH2D2A*’s zTPM levels with those of its neighbouring genes across all 220 Bulk-ECexplorer datasets suitable for this analysis.

**Fig. 6:**
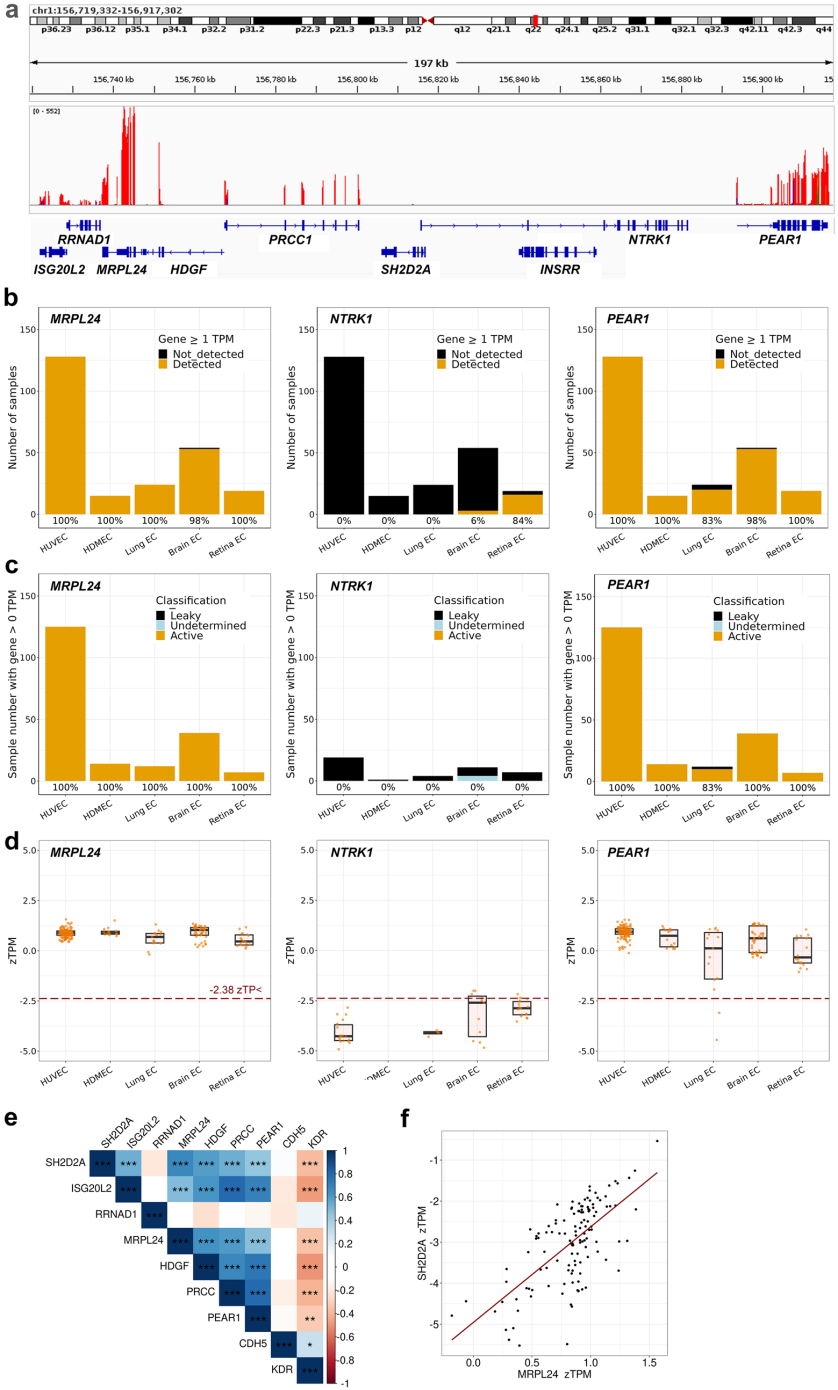
*SH2D2A* expression correlates with neighbouring gene expression in ECs. **a** Read coverage for *SH2D2A* and its neighbouring genes (± 100 kilo base pairs) on chromosome 1 in the HUVEC sample SRR1640083 that has 0.95 TPM for *SH2D2A*; human genome build (hg) 38; Y axis axis: read coverage range 0–575. **b** Stacked bar charts show the number of Bulk-ECexplorer datasets (n = 240) in which the indicated genes were detected above or below the 1 TPM threshold. The percentage of datasets in which the indicated gene was expressed above the threshold is reported for each EC subtype below each bar. **c** Stacked bar charts show the number of Bulk-ECexplorer datasets in which the indicated genes were classified with the GMM method as actively expressed (gold), leakily expressed (black) or in which the gene was not classified (undetermined, blue). Only datasets with a bimodal distribution of gene expression values and detectable transcript were examined; *MRPL24* n = 197, *NTRK1* n = 42, *PEAR1* n = 195. The percentage of datasets in which each gene was classified as actively expressed is reported for each EC subtype below each bar. **d** Range of zTPM values for the indicated genes in Bulk-ECexplorer datasets, including boxplots to illustrate the interquartile range. Each data point represents one dataset; values are shown together with boxplots to illustrate the interquartile range (for TPM values and n, see corresponding source data file). The dashed red line indicates the -2.38 zTPM threshold in HUVEC. Each data point represents one sample; *MRPL24* n = 219, *NTRK1* n = 52, *PEAR1* n = 219 samples across all EC subtypes for each gene (only datasets with gene detection > 0 TPM and without a unimodal distribution were included). **e** Correlation of zTPM values for *SH2D2A* to neighbouring HE genes as well as KDR and CDH5 across all EC types; Pearson’s correlation (r) with Holm adjusted p-values, * < 0.05, ** < 0.01, *** < 0.001; n = 126 (except RRNAD1, n = 125); unimodal samples were excluded. **f** Linear regression of *SH2D2A* zTPM values onto *MRPL24* zTPM values. Each data point represents the zTPM value of one sample (n = 126; unimodal samples were excluded and only samples with detectable *SH2D2A* and MRPL24 expression were included; coefficient of determination R^2^ -0.45; slope co-efficient β_1_ = 2.33 and p-value for regression < 2.2^–16^

The organisation and orientation of the genes surrounding *SH2D2A* are conserved between human chromosome 1 and mouse chromosome 3 (data discoverable at https://genome.ucsc.edu/). Specifically, *SH2D2A* resides between an upstream gene cluster (*ISG20L2, RRNAD1, MRPL24, HDGF* and PRCC) and a downstream gene cluster (*NTRK1* and *INSRR*), both in the human genome and in the mouse genome (not shown). The read distribution in this genomic neighbourhood was mapped for a representative HUVEC sample that contained *SH2D2A* transcripts, which raised the possibility that *SH2D2A* resided at the border between an HE and NE gene cluster in ECs (Fig. 6a). To corroborate this finding across additional HUVEC datasets and further EC subtypes, we used the Bulk-ECexplorer to examine the expression characteristics of transcripts in the *SH2D2A* genomic neighbourhood. Thus, the upstream genes *ISG20L2, RRNAD1* (also known as *METTL25B*), *MRPL24, HDGF* and *PRCC* were consistently reported as HE genes expressed above the 1 TPM threshold (> 99% of EC datasets, Figs. 6b, Supplemental Fig. S5, Supplemental Table 1). The GMM and zTPM methods also classified these upstream genes consistently as actively expressed (> 99% of EC datasets; Figs. 6c, d; Supplemental Fig. S5, Supplemental Table 1). The downstream *PEAR1* gene (Fig. 6a) that resides within 100 kb of the *SH2D2A* transcription start site (TSS) was also classified as HE and actively expressed in > 99% of EC datasets (Fig. 6b–d; Supplemental Table 1). By contrast, the immediate downstream neighbours of *SH2D2A*, *NTRK1* encoding the neurotrophin receptor TRK-A, and *INSRR* encoding the urogenital insulin-related receptor, were only rarely detected in the Bulk-ECexplorer datasets (Fig. 6a; Supplemental Figure S5, Supplemental Table 1), thus identifying them as NE genes in ECs. When datasets contained some transcripts for these genes, the GMM and zTPM methods classified *NTRK1* as not actively expressed and *INSRR* mostly as not actively expressed in all EC subtypes (Fig. 6b–d; Supplemental Figure S5, Supplemental Table 1). The presence of *INSRR* in brain EC datasets may reflect neural cell contamination of primary ECs (see Discussion in [35]). The genomic neighbourhood of *SH2D2A* therefore resembles that of LE genes in ECs, in analogy to a  prior study, in which LE genes were found to reside between HE and NE gene clusters in immune cells [67].

We next correlated the expression levels of *SH2D2A* with those of its neighbouring HE genes across 220 human and mouse Bulk-ECexplorer datasets, again using the standardised zTPM unit to exclude that gene expression might appear correlated only because of global variations in transcript levels between individual datasets. We found that *SH2D2A* expression significantly and positively correlated with the expression of all but one of its HE upstream neighbours across the 220 datasets (*SH2D2A* correlation with *ISG20L2 r* = 0.53, *MRPL24 r* = 0.67, *HDGF r* = 0.59, *PRCC r* = 0.54; Fig. 6e). A less strong, but nevertheless significant, correlation was also observed between the expression of *SH2D2A* and its downstream HE neighbour *PEAR1* (*r* = 0.45; Fig. 6e). In particular, linear regression analysis revealed that variation in *MRPL24* expression could account for 44.9% of the variation in *SH2D2A* expression amongst datasets in the Bulk-ECexplorer (Fig. 6e, f). By contrast, *SH2D2A* expression did not positively correlate with the expression of two other VEGF-induced vascular permeability signalling-relevant HE genes in unlinked genomic loci, *CDH5* (chromosome 16; r = 0.10) and *KDR* (chromosome 4; r = −0.38) (Fig. 6e). Taken together, low level *SH2D2A* expression correlates with genomic proximity to neighbouring, actively expressed genes in ECs, as previously described for leakily expressed genes in other cell types.

### The *SH2D2A* promoter resides within closed chromatin in ECs

**Fig. 7:**
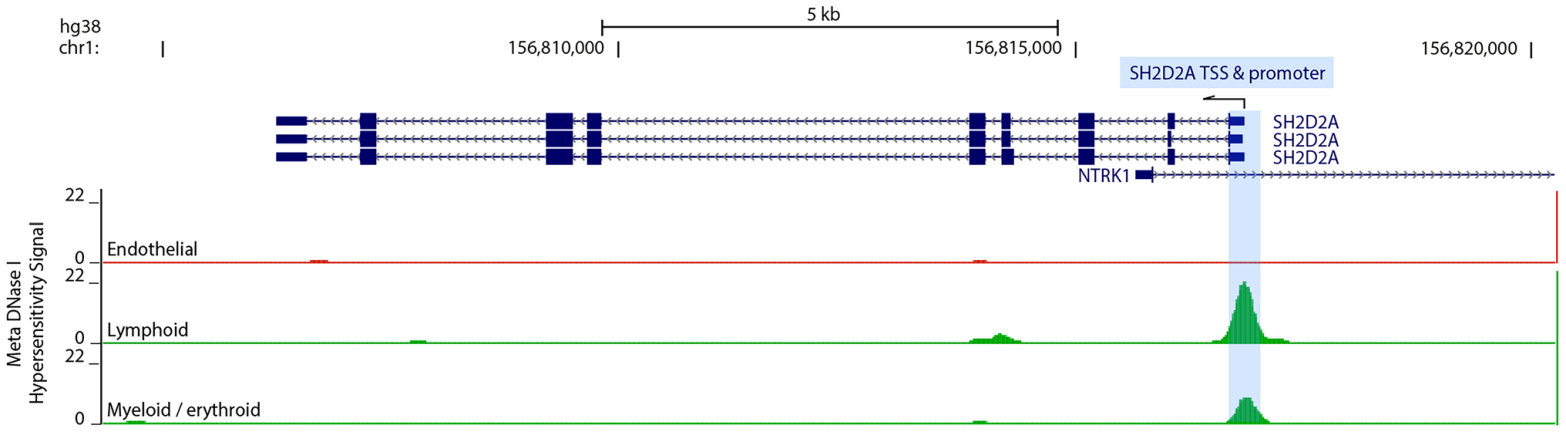
The *SH2D2A* promoter resides in closed chromatin in ECs. The ENCODE meta-DNase I HS signal for the human *SH2D2A* locus is shown for endothelial, lymphoid and myeloid cells (n = 15 samples for each of the three meta-profile). The intron-exon structure of 3 known *SH2D2A* transcripts and the first exon and intron of the neighbouring *NTRK1* transcript are shown. The *SH2D2A* transcription start site (TSS) and promoter region are highlighted in blue.

As chromatin accessibility to transcription factors is an inherent feature of active genomic elements, the promoters of actively expressed genes are accessible also to DNase, and DNase I hypersensitivity sites (DNase HS) can be mapped across a genome, as reported for the human genome in the ENCODE project [75]. In phase 3 of the ENCODE project, a total of 733 human samples were analysed by DNase-seq [45]. Further, the DNase-seq signal profile for 15 samples each for ECs, lymphoid and myeloid/erythroid cells was averaged to provide a DNase HS meta-profile that could be directly compared for accessible chromatin sites in these cell types [45]. By re-analysing these published meta-DNaseq HS data, we found that a strong DNase HS signal encompassed the *SH2D2A* promoter and transcription start site (TSS) in immune cells, as expected, whereas no obvious DNase HS signal was detected at the *SH2D2A* promoter/TSS in ECs, including in HUVECs, HDMECs and lung ECs (Fig. 7). These data show that the *SH2D2A* promoter resides within open chromatin in immune cells but within closed chromatin in ECs. *SH2D2A* therefore has the chromatin hallmarks of a gene that is not actively expressed in ECs, consistent with the gene expression characteristics we have described above.

### Mass spectrometry studies do not report TSAd (SH2D2A) protein in ECs except in rare HUVEC samples with an immune signature

We next examined which vascular permeability regulators were present in publicly available EC proteomes. Having surveyed the ProteomeXchange database (https://www.proteomexchange.org) for suitable EC mass spectrometry datasets (see methods), we retrieved 12 datasets with 52 independent proteomes derived from whole cell lysates or the cytoplasmic/transmembrane fractions of HUVECs as well as whole cell lysates from primary ECs across 7 organs. Since each proteomic dataset was obtained with quantitative approaches that differed in terms of estimation, sensitivity and specificity, we reported protein expression in a binary outcome of ‘detected’ or ‘not detected’.

As *SH2D2A* had been classified as actively expressed in only a minor proportion of EC bulk RNAseq datasets that were mostly derived from HUVECs (see above), we initially examined six proteome datasets from HUVEC whole cell lysates with a range of recorded proteins that spanned from 8210 to 4207 [76–82]. The detection of 6/9 pre-defined core EC markers, including KDR and/or CDH5 (see methods), confirmed cell identity (Supplemental Table 3). Notably, TSAd and SDC2 were not present in the list of reported proteins in any of the 18 samples contained in these datasets (Table 3). By contrast, all other components of the VEGF hyperpermeability pathway examined were recorded across the 7 samples from the largest, 8210-, 8198- and 6876-protein HUVEC datasets, whereas most of them were detected across the 4 samples from the 6678- and 4591-protein HUVEC datasets and half of them in the 7 independent samples from the smallest, 4207-protein HUVEC dataset (Table 3). Thereafter, we extended analysis to whole cell proteomes from ECs of vascular permeability-relevant organs, including the heart [83], lung [79, 84, 85], retina [86], skin [87], brain and kidney [79]. These datasets recorded most components of the VEGF hyperpermeability pathway, including SDC2 in 1/1 skin and 3/3 heart EC samples, but all organ EC proteomes lacked TSAd (Table 3). Finally, we examined whether VEGF stimulation increased TSAd detection, taking advantage of a published proteomics dataset from VEGF-treated versus control human lung ECs [85]. However, TSAd was also not detected in any VEGF-stimulated samples, similar to the controls (Supplemental Table 4). Although the above HUVEC and organ EC proteomes all lacked TSAd, 2/2 T cell samples [88] contained abundant reads for TSAd (Table 3), as expected [27].

We also examined the recorded list of 7625 proteins derived from a dataset of 7 independent HUVEC samples enriched for cytoplasmic and transmembrane proteins at the expense of secreted and nuclear proteins [46], as this approach potentially confers higher detection sensitivity for signal transducers. Similar to the HUVEC whole cell proteome analysis, all permeability pathway-relevant proteins examined were recorded in 4 out of 7 enriched HUVEC samples and most of them were recorded in the other 3 enriched HUVEC samples, except TSAd and SDC2 (Supplemental Table 5). Specifically, SDC2 was recorded in only 1 and TSAd in only 2 of the 7 HUVEC enriched samples; notably, the 2 HUVEC samples that recorded TSAd also recorded immune cell markers (Supplemental Table 3). To more objectively assess the cell type signature of the two HUVEC samples that reported TSAd in the enriched dataset, we first identified proteins exclusive to these two samples and then used them for overrepresentation analysis against the cell type signature gene sets from the Human Molecular Signatures Database, using all the proteins detected across the 7 HUVEC samples of the enriched dataset as the background universe (see Methods). This analysis demonstrated a significant enrichment of a foetal lymphoid cell signature (see methods; Supplemental Table 6). In the same study, 1/3 normal human dermal fibroblast (NHDF) and 3/3 human peripheral blood mononuclear cell (PBMC) cytoplasmic protein-enriched samples also recorded TSAd (Supplemental Table 5). Unexpectedly, these non-EC proteomes also contained EC markers (Supplemental Table 3). It is not known why 12/13 of cytoplasmic fraction-enriched samples in this study contained markers typical of the other cell types examined.

In summary, TSAd was not detected in 27/27 organ EC proteomes examined, and detected in just 2/21 HUVEC samples, both derived from a single dataset in which TSAd expression was linked to an unexpected immune signature. Proteomic data therefore agree with epigenomic data to show that TSAd is robustly expressed in T cells and PBMCs but is not detected in organ ECs, and is typically also absent from HUVEC proteomes.

## Discussion

Here, we have mined publicly available sc-RNAseq, bulk RNAseq, epigenomic and proteomic data to investigate whether components of several proposed signalling axes for VEGF-induced vascular permeability induction are expressed in ECs of organs clinically affected by VEGF-induced oedema or in EC subtypes used for discovery science to elucidate vascular permeability signalling pathways. We found that all signal transducers examined were expressed in the ECs of the organs examined, with organ selectivity for some components such as SDC2, but very low or complete lack of TSAd detection. As these findings are incompatible with a role for TSAd in vascular permeability signalling, we discuss below the evidence and potential limitations of our investigation for each method we employed.

We found that sc-RNAseq datasets of ECs from such human organs consistently contained transcripts for all VEGF permeability pathway-relevant signal transducers examined, with exception of transcripts for TSAd (Fig. 1, Tables 1 and 2). Moreover, TSAd transcripts were also lacking from all EC transcriptomes contained in the EC atlas and barely present in all other EC transcriptomes included in *Tabula Muris* (Fig. 2, Tables 1 and 2). We also used the Bulk-ECexplorer as a complementary resource to determine whether relevant signal transducers were expressed in ECs, and to predict whether detectable gene expression was likely at functional levels [35]. Transcripts for VEGFR2 and CDH5 are defining features of Bulk-ECexplorer datasets, and transcripts for YES1 and SRC are detected as actively expressed genes in these datasets [35]. Except for *SH2D2A* encoding TSAd, all other VEGF permeability pathway-relevant signal transducers examined were consistently and robustly detected, and, accordingly, predicted to be actively expressed and therefore functional (Figs. 3, 4, 5, Supplemental Table 1). By contrast, *SH2D2A* detection in half of the EC datasets of the Bulk-ECexplorer datasets was low and mostly in HUVECs (Figs. 3a, c, S1B), agreeing with prior work in HUVECs, which reported that *SH2D2A* detection in reverse transcribed mRNA required a large number (45) of polymerase chain reaction (PCR) cycles [22]. Moreover, as reported for porcine aortic endothelial (PAE) cells [22], *SH2D2A* transcript levels did not increase in HUVECs after VEGF stimulation (Supplemental Table S2).

Low or absent *SH2D2A* detection in both EC single cell and bulk RNAseq data is incompatible with TSAd’s proposed central role in VEGF/VEGFR2-mediated vascular permeability signalling. For this reason, we considered technical parameters that could have, conceivably, impacted our analysis. Unsuitable sc-RNAseq methods could not account for a lack of *SH2D2A* transcript detection, because detection was robust in immune cells (Fig. 2). For bulk RNAseq analysis, we considered whether the HISAT2-Stringtie pipeline used to quantify transcript abundance in the Bulk-ECexplorer may have impacted *SH2D2A* transcript detection. However, many of the studies from which we sourced EC bulk RNAseq data used alternative pipelines in their own analyses, and inspection of their published summary data similarly indicated absent or very low *SH2D2A* expression (although these studies did not comment on *SH2D2A* detection levels per se). For example, TopHat-Cufflinks was used to process bulk RNAseq data for HDMECs and HUVECs [89], TopHat-HTSeq for retina ECs [90], TopHat-Bowtie for brain ECs [91] and TopHat2-Seqsolve for lung ECs [92]. Further, we considered that insufficient read depth can sometimes explain the absence of a transcript in bulk RNAseq data [61], and, in agreement, datasets with higher read depth were more likely to return reads for *SH2D2A* (Supplemental Fig. S2). However, transcripts detectable only at high read depth are inherently rare and may arise from leaky transcription, as previously observed in many different cell types [62, 69–72], including in ECs [35]. Therefore, we used the GMM and zTPM threshold tools included with the Bulk-ECexplorer to calculate the probabilities of *SH2D2A* being a leaky transcript in ECs. Both methods predicted that *SH2D2A* transcript presence in EC datasets likely reflects leaky transcription (Figs. 4 and 5).

Drawing on the concept of leaky transcription to explain low *SH2D2A* levels in ECs will be controversial, because it implies that TSAd is unlikely to be a central, functional player in vascular permeability signalling as held in current vascular permeability models. For this reason, we have re-considered the strength of evidence that supports a two-component transcriptome that is represented by a bimodal distribution of transcript abundance in bulk RNAseq data and  reflects the leaky versus active transcriptome. Supporting this concept, several studies in different cell types reported that LE transcripts encode proteins not expected to be functional in that cell type; for instance, olfactory-related LE transcripts were identified in cervical carcinoma-derived HeLa cells [70], whereas T helper cell (Th) 1-specific LE transcripts were identified in Th2 cells [73]. In analogy, the T cell marker *CD4* was detected at very low levels in approximately half of the 240 EC RNAseq datasets included in the Bulk-ECexplorer (Fig. 3), which was classified as leaky expression (Figs. 4, 5), akin to prior observations for the erythroid marker *KLF1* in ECs [35]. Further, the expression profiles of *SH2D2A*, *CD4* and *KLF1* were similar to several other genes not expected to be functional within ECs, including other immune genes and osteoblast or sex cell-specific genes [35].

Analysis of epigenomic data across different cell types has previously provided additional and decisive support for the concept of a two-component transcriptome model arising from leaky and active gene expression. For example, a comparison of DNase-seq and RNAseq data for Th2 cells revealed that the activating histone modification H3K9/14ac is enriched near genes classified by the GMM approach as HE/active, but this modification was largely absent from genes classified as LE/leaky [73]. In another example, a study analysing ENCODE RNAseq and ChIP-seq data reported that the promoters of GMM-classified HE/active genes are consistently associated with active chromatin markers in several different cell types, whereas the promoters of GMM-classified LE/leaky genes are frequently associated with repressive chromatin markers in the same cell types [62]. Here, we have also examined ENCODE epigenetic data obtained by DNase-seq and found that the human *SH2D2A* promoter was positioned within open chromatin in 15 leukocyte datasets but within closed chromatin in 15 EC datasets (Fig. 7), including in HUVECs and HDMECs that have previously been reported to contain TSAd protein. This finding is most consistent with *SH2D2A* being an immune-related gene that is either not expressed in ECs or is expressed as a leaky gene in ECs.

Agreeing with the idea that TSAd is a non-expressed or leaky gene in ECs, TSAd was not recorded in 53/55 EC proteome samples examined and the 2 remaining samples that recorded TSAd were HUVECs also enriched in other immune cell markers (Table 3, Supplemental Tables 3 and 6). By contrast, 2/2 T cell and 3/3 PBMC proteome samples examined detected TSAd (Table 3, Supplemental Table 5), thus arguing against the possibility that poor detectability of TSAd peptides accounted for TSAd’s absence in the recorded EC proteomes. Across the EC proteomes examined, our findings link TSAd to immune marker expression rather than a pure EC phenotype. Conceivably, HUVECs may have an inherent propensity for immune cell marker expression under specific environmental conditions. Alternatively, we need to consider the possibility of immune cell contamination, because these samples were part of a study that directly compared endothelial, PBMC and fibroblast proteomes, and several samples from these cell types contained markers of the other cell types included in the study (Supplemental Table 3).

Aside from the finding that HUVEC samples from one laboratory had an immune signature that included TSAd, two other laboratories have reported that TSAd is detectable by immunoblotting in HUVECs and HDMECs [22, 24, 93]. Immunoblotting differs from mass spectrometry by its reliance on highly specific antibodies for protein detection and is likely more sensitive for detecting low protein levels than mass spectrometry-based methods. Therefore, we cannot exclude that TSAd protein could become detectable in EC proteomic datasets generated with technologies that have increased sensitivity for detecting and reporting proteins translated from rare transcripts. Nevertheless, transcripts or protein detected at very low levels may not be of biological significance [62, 70–73]. Furthermore, it is surprising that a protein whose transcripts are either not detectable or detected at levels below 1 TPM in HDMECs (Fig. 3) should be detectable by Western blotting in these cells. Therefore, we have considered that leaky transcripts may not be uniformly distributed across a cell population, but instead can be present in a very small proportion of cells within the overall cell population, as reported for the *Tbx21* gene in Th2 cells [73]. If a very small proportion of ECs were to express TSAd at high enough levels to lead to protein production detectable with very sensitive methods, a hypothetical scenario arises in which rare TSAd-expressing ECs might selectively respond to VEGF with vascular permeability signalling. For example, it has been proposed that vascular leakage occurs at distinct focal points of a blood vessel [94]. However, the sc-RNAseq dataset from human skin, the only human organ reported to contain vessel-associated TSAd protein detectable by immunostaining, lacked an obvious population of *SH2D2A*-positive ECs (Fig. 1). As our analysis of human dermis and trachea as well as mouse organ sc-RNAseq data did not identify a subset of *SH2D2A*-positive ECs in any of these organs, it is difficult to conceive how focal activity of an EC subset that is too rare to be identified by sc-RNAseq could then cause measurable vascular leakage across an organ.

We have also considered whether published immunostaining data for TSAd support the possibility of an EC subset with functional TSAd protein levels. However, we found that no prior study was compatible with the existence of TSAd hotspots in vascular endothelium. Firstly, one study [28] found TSAd lacking from blood vessels in all human organs examined except in the dermis, where continuous staining of putative vessels was reported. Secondly, another immunostaining study suggested continuous staining of kidney endothelium [22]. However, it should also be considered that neither immunostaining study had specifically validated the EC identity of the stained cell type (as opposed to an endothelium-associated cell type) nor have prior studies excluded cross-reactivity of the antibody with an EC epitope that does not belong to TSAd, even though antibodies were clearly shown to be specific for TSAd in immune cells. In agreement with antibody cross-reactivity to a non-TSAd epitope in ECs, the supplemental data of a prior study showed positive reactivity in the lung lysates of constitutive TSAd-null mice by immunoblotting [95]. Additional evidence from more informative immunostaining protocols, immunoblotting with conclusively validated antibodies for ECs and/or the analysis of cell-type specific TSAd knockout mice would therefore be required to uphold an EC cell autonomous role for TSAd in vascular permeability. Alternatively, it should be considered that TSAd expression in non-ECs, such as vessel-associated immune cells, contributes to EC barrier regulation to explain impaired VEGF-induced vascular permeability induction in TSAd knockout mice, or that, in such genetically-modified mice, another genomic element was inadvertently disrupted during gene targeting to affect vascular barrier function, such as, hypothetically, a regulatory element for one of the HE genes in the *SH2D2A* neighbourhood. Notably, *SH2D2A* neighbouring genes have not yet been studied for potential functions in vascular permeability, although its closest genomic neighbour, HDGF, has been described as an angiogenesis regulator [96, 97].

In summary, EC expression of TSAd transcripts is either not detectable, or detectable only at very low levels by RNAseq assays. When combined with EC epigenomic and proteomic data, these findings support the conclusion that TSAd is either not expressed, or a leaky and non-functional gene in ECs. By extension, our findings also argue against a central role for TSAd in the endothelial vascular permeability signalling cascade, thereby excluding it as useful target for therapeutic approaches that seek to inhibit VEGF-induced oedema. By contrast, all other genes implicated in VEGF-induced permeability signalling were robustly expressed in ECs at both transcript and protein level, with some organ specificity. These findings should be considered in studies seeking to identify therapeutic tools for modulating vascular barriers. Further, our study may provide the impetus for future work to examine vascular permeability-relevant gene expression in additional contexts, such as tumour biology, lung and eye disease, where vascular permeability mediators other than VEGF contribute to vascular barrier breakdown.

## Supplementary Information

Below is the link to the electronic supplementary material.

Supplementary file1 (XLSX 9052 KB)

Supplementary file2 (PDF 1144 KB)

## Data Availability

No datasets were generated or analysed during the current study.
