# Supplementary material for: Endothelial transcriptomic, epigenomic and proteomic data challenge the proposed role for TSAd in vascular permeability
Source: Angiogenesis. 2025 Mar 13;28(2):21. doi: 10.1007/s10456-025-09971-x (PMC11906500; doi:10.1007/s10456-025-09971-x)
Supplement: Supplementary file 2 — Supplementary file2 (PDF 1144 KB) [file 10456_2025_9971_MOESM2_ESM.pdf]

Supplemental tables

(all tables are provided as tabs in an excel file)

Supplemental Table 1

TPM, GMM and zTPM data for VEGF-induced vascular permeability pathway genes.

Summary table listing TPM, GMM and zTPM data for genes implicated in VEGF-induced vascular permeability signalling, the T cell marker *CD4* and genes in the immediate *SH2D2A* genomic neighbourhood.

Supplemental Table 2.

TPM data from VEGF-stimulated HUVECs and unstimulated controls for VEGF-induced vascular permeability pathway genes.

Gene expression levels in TPM and differential expression analysis results (log2FoldChange and adjusted P-value) are shown for VEGF-induced vascular permeability pathway genes in 2D-cultured (PRJNA807293, PRJNA431557) and spheroid-cultured (PRJNA945485) HUVECs. ProteomeXchange identifiers and PubMed IDs (dataset) and cell type, including EC subtype and culture method (cell type) are indicated. Log2FC = Log2FoldChange.

Supplemental Table 3

Detection of selected EC, immune and mesenchymal markers in proteomic datasets scored for VEGF hyperpermeability pathway proteins.

The table summaries whether the indicated proteins were detected (D) or not detected (ND) in individual samples from 12 publicly available human and mouse datasets obtained by mass-spectrometry of whole cell lysates and three publicly available human datasets obtained by mass-spectrometry after enrichment for cytoplasmic proteins. Only samples from healthy, untreated conditions were scored; HUVEC data were derived from cultured cells. The table also lists the ProteomeXchange identifiers and PubMed IDs (dataset), species (Hs = *Homo sapiens* or Mm = *Mus musculus*), cell type, including EC subtype, number of detected proteins (proteins), number of independent samples per dataset (sample) and whether the detection of the indicated protein markers is consistent with an EC, immune cell (IC) and/or mesenchymal cell (MC) signature (signature).

Supplemental Table 4.

Detection of VEGF vascular permeability pathway proteins in whole cell lysates of VEGF-stimulated HUVECs and controls.

The table summaries whether the indicated proteins were detected (D) or not detected (ND) in control or VEGF-stimulated HUVEC samples in the dataset PXD029834. The table lists the ProteomeXchange identifiers and PubMed IDs (dataset), species (Hs = *Homo sapiens*), cell type (including EC subtype), number of detected proteins (proteins), number of independent samples per dataset (sample) and whether the proteome includes EC, immune cell (IC) and/or mesenchymal cell (MC) markers (cell type signature defined by key markers presented in Supplementary Table 3).

Supplemental Table 5

Detection of VEGF vascular permeability pathway proteins after enrichment for cytoplasmic proteins.

The table summaries whether the indicated proteins were detected (D) or not detected (ND) in three publicly available datasets (PDX003406, PDX003412 and PXD001416) of three human (Hs = *Homo sapiens*) cell types including HUVECs, normal human dermal fibroblasts (NHDFs) and peripheral blood mononuclear cells (PBMCs). The table lists ProteomeXchange identifiers and PubMed ID (dataset), the number of detected proteins (proteins), number of independent samples per dataset (sample) and whether the proteome includes EC, immune cell (IC) and/or mesenchymal cell (MC) markers (cell type signature defined by key markers presented in Supplementary Table 3).

Supplemental Table 6.

TSAd-expressing HUVEC samples in PDX003406 have an immune signature.

Comparison of cell type signatures specific to TSAd-expressing (2/7) versus non-TSAd-expressing (5/7) HUVEC samples in PDX003406, a dataset enriched for cytoplasmic proteins. The 'Gene set' column lists the name of the cell type signature gene sets from the Molecular Signatures Database. The 'Gene ratio' column indicates how many proteins encoded by genes exclusive to TSAd-expressing samples are present in each gene set. The 'Background ratio' column indicates how many genes coding for proteins detected across all samples are present in each gene set. The columns listing the P value and adjusted P value, respectively, are followed by the ‘Gene set constituent genes’ column that lists the names of the genes included in each gene set that are exclusive to the 2 TSAd-expressing samples.

Supplemental figures

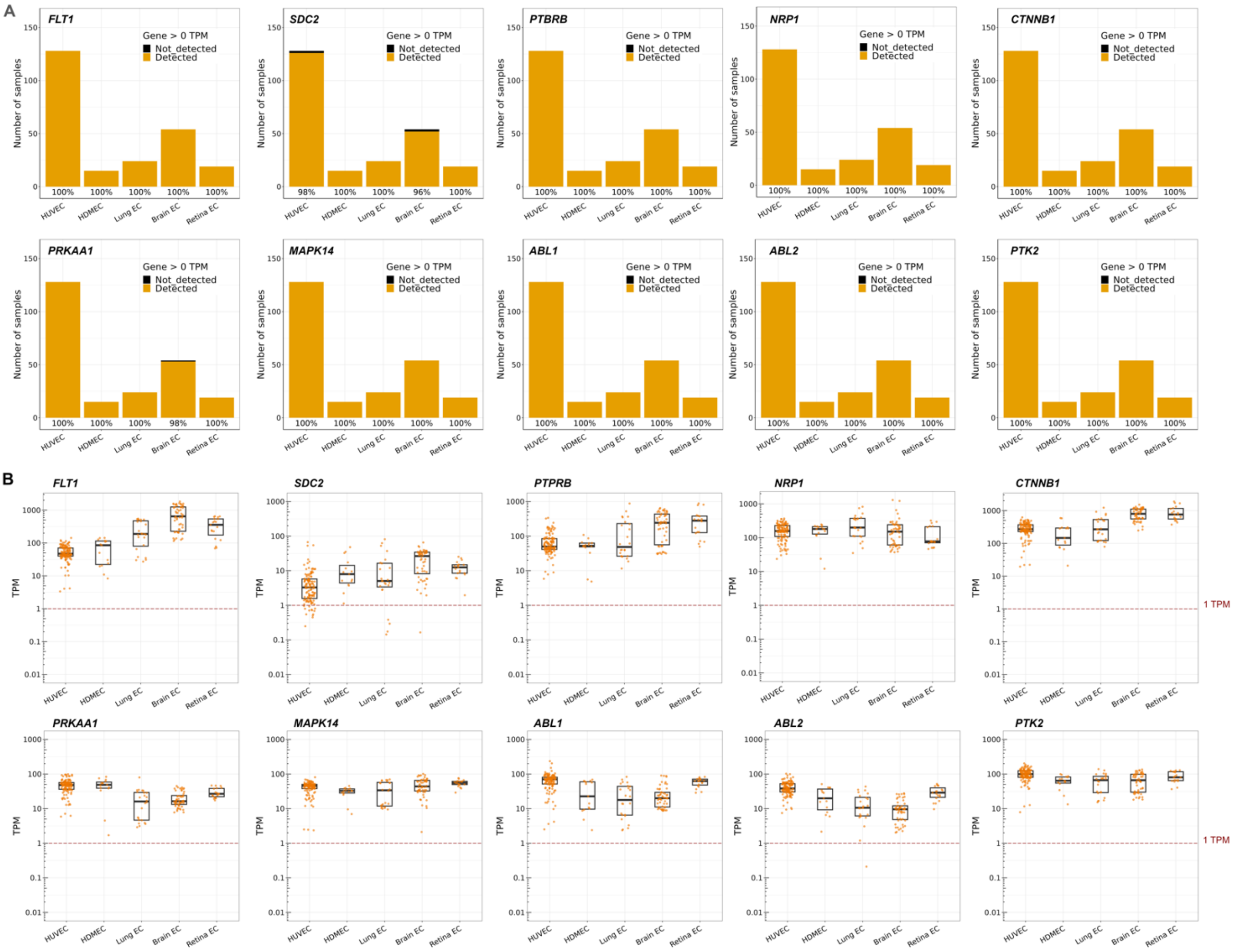

Supplemental Figure S1

Detection rates and transcript levels for key signal transducers implicated in VEGF-induced vascular permeability signalling.

(**A**) Stacked bar charts indicating the number of datasets in which genes were detected (> 0 TPM) or not detected (0 TPM) for HUVEC n = 128, HDMEC n = 15, mouse lung EC n = 24, mouse brain EC n = 54, mouse retina EC n = 19 (total n = 240). Detection frequencies are shown as percentages below the bars for each EC subtype.

(**B**) Detection levels for genes with expression > 0 TPM, including boxplots with the median (centre line) and interquartile range (box limits); each data point corresponds to the value for one dataset; the red dashed line indicates the 1 TPM threshold; see corresponding source data file for n.

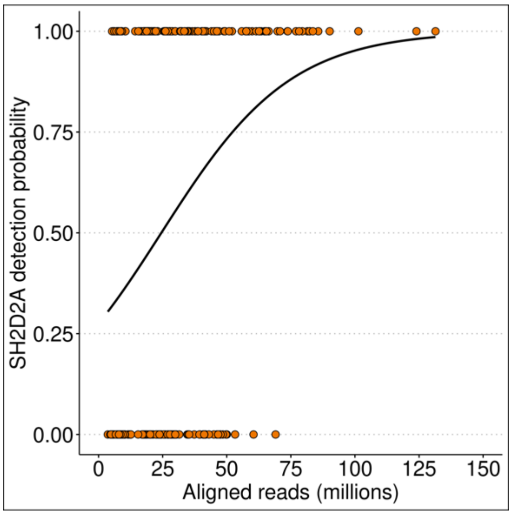

Supplemental Figure S2

Association between the number of aligned reads and *SH2D2A* transcript detection.

A logistic regression model, with the binary dependent variable representing *SH2D2A* detection (1 = detected, 0 = not detected) and the continuous predictor being the number of aligned reads; the black curve represents the logit(probability) of detecting *SH2D2A* with an increased number of reads.

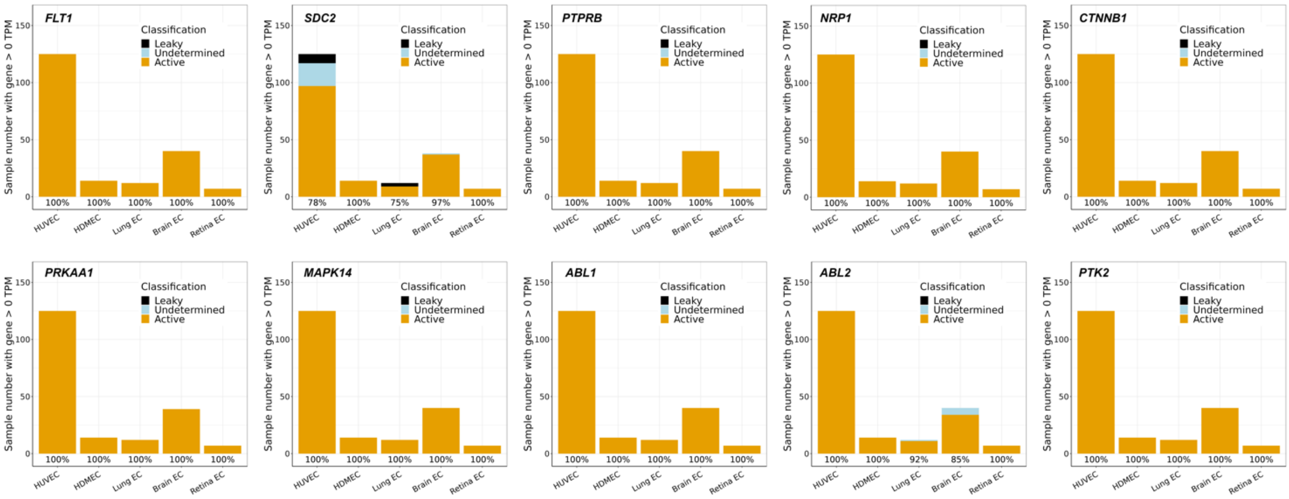

Supplemental Figure S3

GMM-based active versus leaky classification of genes implicated in VEGF-induced vascular permeability signalling.

Stacked bar charts indicate the number of datasets in which genes were classified as active, leaky, or undetermined for each EC subtype. The percentage of datasets in which each gene was classified as active in each EC subtype is reported below the corresponding bar (n = 198 for all genes except PRKAA1 n = 197).

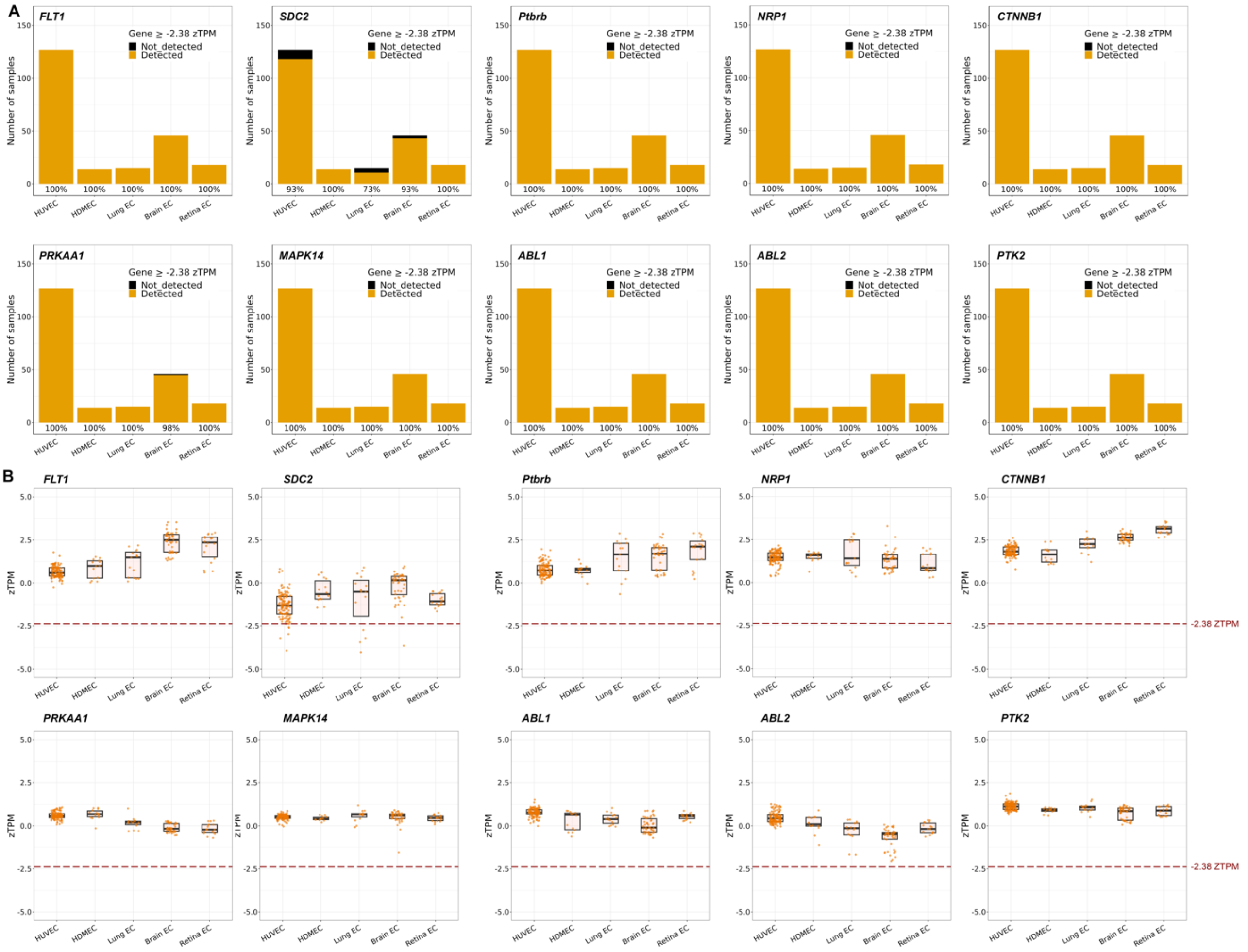

Supplemental Figure S4

Active versus leaky classification of genes implicated in VEGF-induced vascular permeability signalling after zTPM standardisation.

(**A**) Stacked bar charts indicate the number of datasets in which genes were detected over or under the zTPM threshold for HUVEC (-2.38), resolved by EC subtype; the percentage of datasets ≥ -2.38 is indicated below each bar (n = 220 for all EC subtypes together).

(**B**) For genes with expression > 0 TPM, zTPM values are shown as individual datapoints for each dataset in each EC subtype, including boxplots with the median (centre line) and interquartile range (box limits); the red dashed line indicates the -2.38 zTPM threshold for HUVEC; see corresponding source data file for n.

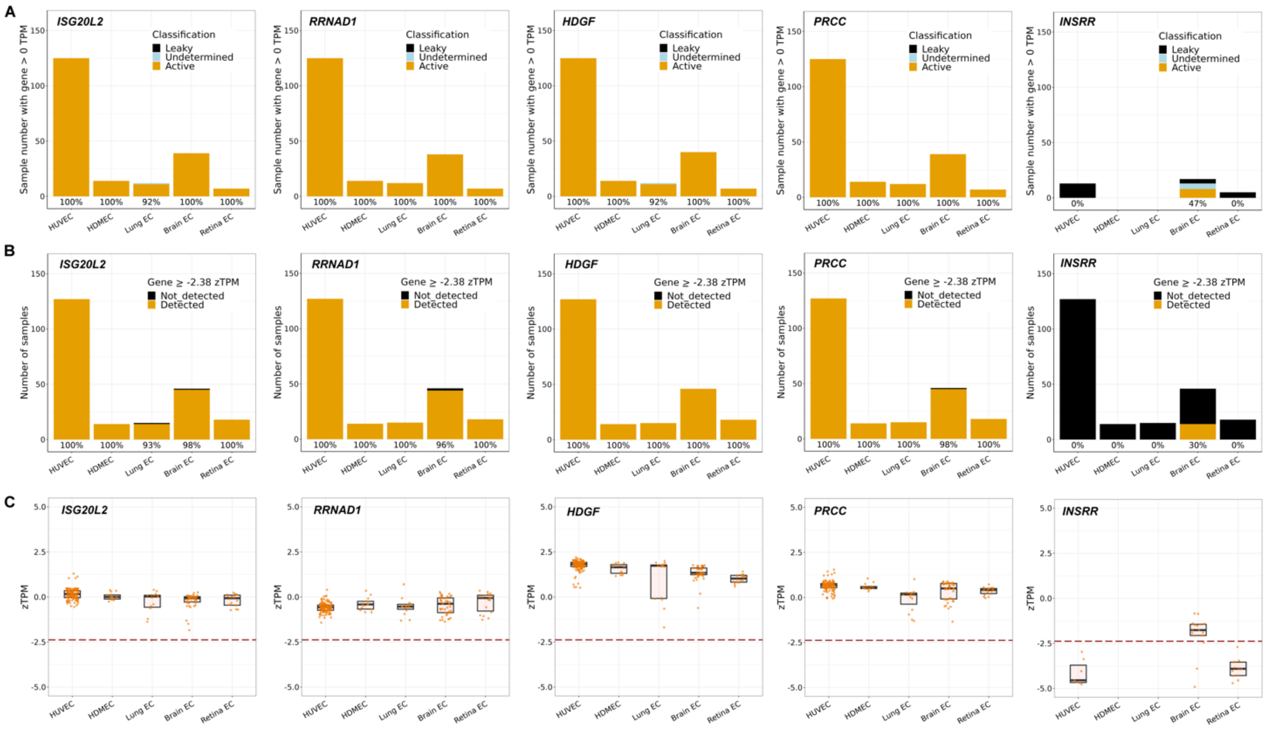

Supplemental Figure S5

Active versus leaky expression of SH2D2A neighbouring genes in mouse and human ECs bulk RNAseq data from the BulkECexplorer.

(**A**) Stacked bar charts indicating the number of datasets in which the indicated genes were classified as active, leaky, or undetermined in each EC subtype with the GMM method; the percentage of datasets in which each gene was classified as active in each EC subtype is reported below each bar.

(**B**) Stacked bar charts indicating the number of datasets in which the indicated genes were detected over or under the -2.38 zTPM threshold for HUVECs, indicated with a red dashed line; for each EC subtype, the percentage of datasets with detection over the threshold is indicated below each bar.

(**C**) For genes with detection levels > 0 TPM, the zTPM value for each dataset is shown as an individual datapoint for each EC subtype, including boxplots with the median (centre line) and interquartile range (box limits).

(**A-C**) See corresponding source data file for n.
